# Neutrophil Extracellular Traps Delay Diabetic Wound Healing by Inducing Endothelial-to-Mesenchymal Transition via the Hippo pathway

**DOI:** 10.7150/ijbs.78046

**Published:** 2023-01-01

**Authors:** Shuofei Yang, ShuangShuang Wang, Liang Chen, Zheyu Wang, Jiaquan Chen, Qihong Ni, Xiangjiang Guo, Lan Zhang, Guanhua Xue

**Affiliations:** 1 Department of Vascular Surgery, Renji Hospital, School of Medicine, Shanghai Jiao Tong University, Pujian Road 160, Shanghai 200127, China; 2 Songyuan Central Hospital, Songyuan Children's Hospital, Songyuan, China

**Keywords:** neutrophil extracellular traps, diabetic foot ulcer, wound healing, endothelial-to-mesenchymal transition, Hippo pathway

## Abstract

Diabetic foot ulcers (DFUs) are among the most frequent complications of diabetes with significant morbidity and mortality. Diabetes can trigger neutrophils to undergo histone citrullination by protein arginine deiminase 4 (encoded by *Padi4* in mice) and release neutrophil extracellular traps (NETs). The specific mechanism of NETs-mediated wound healing impairment in diabetes remains unknown. In this study, we show neutrophils are more susceptible to NETosis in diabetic wound environments. Via *in vitro* experiments and *in vivo* models of wound healing using wide-type and *Padi4*^-/-^ mice, we demonstrate NETs can induce the activation of PAK2 via the membrane receptor TLR-9. Then PAK2 phosphorylates the intracellular protein Merlin/NF2 to inhibit the Hippo-YAP pathway. YAP binds to transcription factor SMAD2 and translocates from the cytoplasm into the nucleus to promote endothelial-to-mesenchymal transition (EndMT), which ultimately impedes angiogenesis and delays wound healing. Suppression of the Merlin/YAP/SMAD2 pathway can attenuate NET-induced EndMT. Inhibition of NETosis accelerates wound healing by reducing EndMT and promoting angiogenesis. Cumulatively, these data suggest NETosis delays diabetic wound healing by inducing EndMT via the Hippo-YAP pathway. Increased understanding of the molecular mechanism that regulates NETosis and EndMT will be of considerable value for providing cellular targets amenable to therapeutic intervention for DFUs.

## Introduction

Diabetic foot ulcers (DFUs), among the most complex, costly, and frequent complications of diabetes, can result in significant morbidity and mortality 1. Impaired wound healing in diabetes is attributed to poor circulation, neuropathy, and uncontrolled infections 2. Inflammation is a typical feature of the wound healing process, and neutrophils are recruited early to the wound bed as sentinels of the innate immune system 3. Recently, much interest has focused on neutrophil extracellular traps (NETs), a host defense mechanism to contain and kill pathogens using a sticky extracellular network loaded with bactericidal proteins 4. NETosis, the process of NET formation, begins with the activation of peptidyl arginine deiminase 4 (PAD4, encoded by *Padi4* in mice), which leads to histone citrullination, massive chromatin decondensation, and nuclear localization of granular enzymes 5. High glucose *in vitro* and hyperglycemia in diabetic patients increase the release of NETs 6. Moreover, local DFU infection can trigger neutrophil activation and NET release 7. Excess NETosis is associated with tissue damage 8. We recently reported that NETs were markers of wound healing impairment in patients with DFUs treated in a multidisciplinary setting 9. However, the specific molecular mechanism by which NETs delay diabetic wound healing is undetermined.

Angiogenesis plays a significant role in the skin wound healing. Endothelial cell (EC) homeostasis is crucial for regulating angiogenesis. EC plasticity is represented by the endothelial-to-mesenchymal transition (EndMT) 10. It has been shown that EndMT is associated with decreased angiogenesis during wound healing process 11. NETs can drive EndMT through degradation of VE-cadherin and subsequent activation of β-catenin signaling 12.

The Hippo pathway is an evolutionarily conserved signaling cascade regulating organ size, tissue regeneration, and stem cell self-renewal 13. In mammals, the core module of this pathway consists of the serine/threonine kinases Stk4 (Mst1), the large tumor suppressor homolog 1/2 (Lats1/2) kinases, and their interaction partners MOB kinase activator 1 (MOB1) 14. Activation of Hippo signaling leads to exclusion of Yes-associated protein (YAP), which interacts with transcription factors and regulate gene expression, from the nucleus. The tumor suppressor Merlin, also known as neurofibromatosis type 2 (NF2), functions as an upstream regulator of the Hippo signaling pathway 15. Hippo pathway dysregulation is associated with impairment of angiogenesis and delayed wound healing in diabetes 16. It is unclear whether Hippo pathway can mediate the impaired angiogenesis by inducing EndMT during diabetic wound healing.

Poor understanding of the cellular and molecular mechanisms that delay diabetic wound healing precludes new therapies or drugs. This study aims to explore the regulatory proteins in the Hippo pathway and how NETs regulate EndMT during diabetic wound healing, and search for potential therapeutic targets for DFUs.

## Methods and Materials

### Ethical approval

This study was approved by the institutional review board of Renji Hospital, School of Medicine, Shanghai Jiaotong University (No. 2020-031) on animal experimentations and human samples test. The human studies were in accordance with the principle of the Helsinki Declaration. All the participants provided their informed consent. Consents had been received from patients for reporting individual data.

### Patient study

Four groups of individuals were enrolled: healthy controls, patients with diabetes, patients with non-diabetic ulcers, and patients with DFUs. We collected data on the participants' demographics, comorbidities, laboratory tests, and wound information. Surgical procedures, including revascularizations, wound interventions, and adverse outcomes, were also recorded. The patients were followed under routine ambulatory care for wound treatment. The patients with DFUs underwent digital photographic documentation of their wounds. The diabetic ulcer severity (DUSS) and Society for Vascular Surgery Wound, Ischemia, and Foot Infection (WIfI) scores were calculated for each DFU patient 17, 18.

### Markers of NETs

Nucleosomes were measured with the Cell Death Detection ELISA^PLUS^ kit (Roche, Germany) according to the manufacturer's instructions. Citrullinated histone H3 (CitH3) was determined as previously described 19. In brief, plasma samples were mixed with a monoclonal mouse anti-histone biotinylated antibody in a streptavidin-coated plate. A rabbit histone H3 (ab176842, Abcam) antibody was used in a second phase. Detection was performed with a peroxidase-linked antibody (MBS135301, DakoCytomation). Values were normalized to a pool of samples from normal subjects that was included in all the microplates. Values are expressed as individual absorption values. Cell-free double-stranded DNA (cfDNA) was measured after phenol extraction using a Qubit 2.0 Fluorometer (Thermo Fisher Scientific, Courtaboeuf, France). Tissue extracts from wounds (20μg) were incubated for 1 hour at 37℃ in 200μl of 0.1M HEPES buffer, pH 7.5, containing 0.5M NaCl, 10% dimethylsulfoxide, and 0.1 mM elastase substrate (methoxysuccinyl-ala-ala-pro-val-p-nitroanilide, Calbiochem, Rockland, USA). Substrate degradation was determined by measuring OD_410_ (Dynatech MR5000; Dynatech Labs, Chantilly, Virginia). A standard curve for degradation was prepared using commercial human neutrophil elastase (0.01 to 0.03 μg/ml, with a range of 0 to 0.514 OD units). Results were expressed as elastase concentration/20 μg of protein extract.

### Capture ELISA of MPO-DNA complex

For the capture antibody, 5 μg/ml anti-MPO mAb (ab9535, Abcam) was coated onto 96-well plates (dilution 1:500 in 50 μl) overnight at 4°C. After 3 rounds of rinsing, 20 μl of the samples was mixed with 80 μl incubation buffer containing peroxidase-labeled anti-DNA mAb (Cell Death ELISA^PLUS^, 1:25, Roche, Germany). The plate was incubated for 2 h and shaken at 300 rpm at room temperature. After 3 rounds of rinsing, peroxidase substrate (100 μl) was added. Absorbance at 405 nm wavelength was measured after 20 minutes of incubation at room temperature in the dark. Values of soluble NET formation are expressed as percentage increases in absorbance above the control.

### Animal experiments

Animals used in this study were handled in accordance with the Guide for the Care and Use of Laboratory Animals published by the National Institutes of Health. The mice were purchased from the Shanghai Laboratory Animal Center and housed at the Animal Science Center at Shanghai Jiao Tong University School of Medicine (the mice were maintained under a 12 h light/dark cycle at 22°C, with two mice per cage). All the experiments involving the mice were conducted in accordance with prior approval of the Ethical Review Board. The establishment and assessment protocol of skin wound healing animal models was performed as previously described 20. WT or *Padi4*^-/-^ (genetic background C57BL/6J) mice were made diabetic using streptozotocin (STZ) or left normoglycemic after STZ buffer. Mice (7 to 9 weeks old, n = 6 per group) with random genders were randomly assigned to a treatment group and anaesthetized with isoflurane. All mice were obtained from the animal facility of Shanghai Jiao Tong University School of Medicine.

Two full-thickness excisional wounds were made to the shaved dorsal skin. Full-thickness excisional wounds were made in a midline skin fold using a sterile, disposable 5-mm biopsy punch (Kai Industries, Tokyo, Japan), generating one wound on each side of the midline. The wounds were photographed using an Olympus camera on days 0, 3, 6, and 9 after wounding. The wound areas were calculated as previously described 21. In brief, the wound diameter was measured using Vernier calipers, and the wound area was calculated using a standard formula for the area of an ellipse (semi-major diameter × semi-minor diameter × Pi). The wounds' superficial blood flow was sequentially analyzed by color laser Doppler (Moor, UK), and the ratio of the wounds' blood flow was calculated. The wound size and Doppler analyses were assessed in a blinded fashion. Control oligonucleotides (scramble sequence) and SMAD2 siRNA (1 mg/wound) were delivered topically by pipette into the wound cavity (10 mL in a vehicle of 30% pluronic F-127 gel [it liquifies at 4℃ but solidifies at body temperature], Sigma-Aldrich) immediately after wounding.

### Cells and cell cultures

HUVECs (PCS-100-013, ATCC) were grown in endothelial growth medium 2 (comprising endothelial basal medium 2 with growth factors and other supplements) with 2% fetal bovine serum. The cells were then incubated with neutrophils (control group), NETs (NETs group), NETs treated with 0.1 mg/ml DNase I (InvivoGen, San Diego, CA, USA), NETs and TLR9 antagonist (ODN 2088, San Diego, CA, USA), NETs and PAK2 inhibitor (FRAX597, Selleck Chemicals, Houston, TX, USA), and NETs and Hippo pathway inhibitor (XMU-MP-1, Selleck Chemicals, Houston, TX, USA) for 12 h at 37℃ under 5% CO_2_. Then the cells were fixed with 10% polyformaldehyde for immunofluorescence analysis. Cell lysates were collected for Western blotting.

### Lentivirus-mediated gene expression and cell transfection

Lentiviral vectors (CS-RfA-ETBsd and pENTRE4-H1) were obtained from the RIKEN BRC DNA Bank and injected into human umbilical vein endothelial cells (HUVECs). Culture supernatants containing virus particles were subjected to ultracentrifugation at 50,000 × g for 2 h to obtain concentrated lentivirus preparations. Sequences of short hairpin RNAs (shRNAs) expressed by lentiviral vectors or small interference RNA products are listed in Table S2. pCSC-flag Merlin/NF2 and pCSC2-flag SMAD2 were generated by PCR subcloning from cDNAs synthesized using HUVEC total RNA. Point and deletion mutants of Merlin/NF2 constructs were generated using single-primer-based site-directed mutagenesis 22. A PCR fragment of flagMerSA was subcloned into a Dox-inducible entry vector and then flipped into the SLIK-neo vector using Gateway LR Clonase II Enzyme mix (Invitrogen, Carlsbad, CA). Cloning and mutagenesis were verified by standard DNA sequencing. Primer sequences for mutagenesis are listed in Table S2. Then jetPEI-HUVEC (Polyplus, Illkirch, France) was used to transfect HUVECs with DNA plasmids according to the manufacturer's instructions.

### *In vitro* test of NET release

The MPO-DNA complex was used as a quantified marker of NET release with a capture ELISA. NETs were visualized by immunofluorescence confocal microscopy as previously described 23. The samples were stained using anti-human neutrophil elastase (ab68672, Abcam) and anti-human myeloperoxidase (#556035, BD Bioscience) antibodies. Primary antibodies were detected with the following secondary antibodies: Alexa Fluor 488-conjugated donkey anti-mouse and Alexa Fluor 568-conjugated donkey anti-rabbit (ab150105 and ab175470, Abcam). Visualization was performed with a Nikon ECLIPSE Ti microscope (Tokyo, Japan). The percentage of NET-releasing cells was determined by examining 200 cells with a double-blind experimental procedure.

### NET production and isolation

NETs were prepared and isolated as previously described 24. In brief, 1.5 × 10^6^ *in vitro*-stimulated neutrophils were left in culture for 4 h. Then the medium was removed, and the cells were washed with RPMI. Two milliliters of RPMI were added to each well and NETs were collected on supernatant medium after vigorous agitation. The medium was centrifuged at 20 × g for 5 min and the supernatant phase containing NETs was collected and stored at -20℃. A very low NET concentration collected from untreated control neutrophils that underwent spontaneous NET generation (approximately 3%) was used as a vehicle control. NETs were produced with neutrophils from the patients and healthy controls.

### Neutrophil isolation

The isolation of neutrophils was performed by using Polymorphprep (Axis-Shield, Oslo, Norway) following the manufacturer's protocol. Purity and viability of neutrophil was assessed by Diff-Quik and Trypan blue stain, respectively (both >95%). RPMI 1640 plus 1% FBS was used as the culture medium for all reactions. Purified neutrophils (1 × 10^6^) isolated from healthy controls were incubated for 3 h at 37°C in 5% CO_2_ and then treated with 6% plasma or platelets isolated from ulcer-related arteries and non-ulcer-related healthy vessels of the DFU patients or from the control individuals.

### Subcellular fractionation

Nuclear and cytoplasmic fractions of cell lysates were prepared using NE-PER Nuclear and Cytoplasmic Extraction kits (#78835, Thermo Fisher Scientific, Courtaboeuf, France) according to the manufacturer's instructions. Subcellular fractionation was validated by immunoblotting using anti-lamin (ab16048, Abcam) and anti-a-tubulin (#2144, Cell Signaling) antibodies to identify nuclear and cytoplasmic markers.

### Immunofluorescence

Wound tissue sections for immunofluorescence analysis were prepared as previously mentioned. The sections were then de-paraffinized, rehydrated, and washed in distilled water. Cell slides for immunofluorescence were fixed with paraformaldehyde for 20 min. The slides were washed with PBS three times, permeabilized with 0.1% Triton X-100, and incubated with 10% BSA in PBS to minimize non-specific binding of the primary antibody. They were then incubated with the appropriate primary antibodies overnight at 4℃. After washing, the molecules of interest were visualized by treatment with a secondary antibody and observed under a fluorescence microscope. The nuclei were counterstained with DAPI. All the experiments were performed in triplicate. In addition, Sytox green dyeing was performed to stain the extracellular DNA.

### Immunoprecipitation and immunoblotting

Immunoprecipitation was performed according to the manufacturer's instructions for protein A/G magnetic beads (Biotool, Houston, TX). Briefly, cells were lysed in lysis buffer containing 0.5-1% Triton X-100, 50 mM Tris-HCl, 50-150 mM NaCl, 1 mM EDTA, 0.1 mM PMSF, 10 mM NaF, and 1 mM Na_3_VO_4_. Supernatants were incubated at 4℃ overnight with anti-flag-agarose beads (Sigma, Oakville, ON, Canada). Antibody-bound beads were washed three times with lysis buffer, boiled in SDS-PAGE sample buffer, and subjected to standard immunoblotting. Primary antibodies recognized were PAD4 (mouse monoclonal anti-human), PAD4 (rabbit monoclonal anti-mouse), CD31, H3, histone H3 (citrulline R2 + R8 + R17), VE-cadherin, FSP-1, SLUG, SMAD2, β-actin, flag tag (ab128086, ab214810, ab28364, ab176842, ab5103, ab33168, ab197896, ab27568, ab33875, ab16039, and ab1162, 1:1,000, Abcam), YAP, p-YAP, Lats1, Mst1, MOB1, Taz, Snail-1, PAK2, Merlin, p-Merlin (#14074, #4911, #3477, #14946, #13730, #4883, #3879, #2608, #12888, #13281, 1:1,000, Cell Signaling). For detection, we used secondary antibodies conjugated to horseradish peroxidase, which were rabbit anti-mouse (ab97046, 1:5,000, Abcam) and goat anti-rabbit (ab6721, 1:10,000, Abcam). Detection was developed by enhanced chemiluminescence reactions (#23225, Millipore).

### Quantitative real-time PCR

Total RNA was extracted using RNeasy Mini kits (Qiagen, Mississauga, ON, Canada). For mRNA analysis, cDNA was amplified by quantitative real-time PCR and normalized to 18S ribosomal RNA. Each reaction was performed in triplicate. Quantification was performed by the 2^-ΔΔCt^ method. Pre-optimized primers were obtained from Sigma (KiCqStart Primers).

### Cell functional assays

The following functional assays were performed: bromodeoxyuridine (BrdU) incorporation assay using cell proliferation colorimetric assays (Roche, Germany) and Matrigel assays with HUVECs was performed as previously described using BD Matrigel Basement Membrane Matrix (BD Bioscience, Sparks, MD) 25. For migration assays and plasma membrane capacitance analysis, confluent HUVECs were plated on an electric cell-substrate impedance sensing (ECIS) chip array (8W1E or 8W10E) and the migration speed was calculated in mm/h. The capacitance of the plasma membrane was the capacitance (μF/cm^2^) as reported by Giaever and Keese. Changes in capacitance of the EC membrane reflect structural changes in the plasma membrane and rearrangement of the cytoskeleton (the EC capacitance was ~1.1 mF/cm^2^, whereas for the mesenchymal cells, it was ~3.0 mF/cm^2^) 26.

### PAD4 activity assays

PAD4 activity in lithium-heparin plasma and skin extracts was assessed with commercially available kits (Cayman Chemicals, Ann Arbor, MI, USA). PAD4 activity was normalized based on the protein concentration measured with a bicinchoninic acid-based kit used according to the manufacturer's instructions.

### Histology and image analysis

At days 3 or 6 post-wounding, the wounds were harvested and fixed in 10% buffered formalin (16 h at 4℃, Sigma) for embedding in paraffin. Sections were deparaffinized, rehydrated, and stained with anti-histone H3 (citrulline R2 + R8 + R17) (ab5103, 1:100, Abcam), anti-MPO (Abcam, ab9535; 1:100), anti-CD31 (ab28364, 1:50, Abcam), anti-Snail-1 (#3879, 1:50, Cell Signaling), and SMAD2 (ab33875, 1:100, Abcam). All of the antibodies were used overnight at 4℃. Stained slides were photographed and analyzed in a blinded fashion using a Zeiss LSM 780 confocal microscope (Zeiss, Carl Zeiss, Germany). Vascular density in the wounds was counted after immunostaining for CD31 and α-SMA. Ten fields per section/animal (n = 6; 400 × magnification) were randomly examined and averaged to analyze the number of CD31-positive blood vessels in the wound edges. The images were analyzed using ImageJ software. Vascular density is expressed per square millimeter. EndMT of ECs was assessed by Snail-1 immunostaining combined with CD31 staining.

### Transmission electron microscopy and scanning electron microscopy of wound tissue

Sections of wound tissue were fixed for 2 h in 4% buffered glutaraldehyde. The sections were cut into smaller pieces, postfixed in 1% osmium tetroxide, sequentially dehydrated through graded alcohols, infiltrated through Epon812, and then embedded in resin. Thin sections were cut and stained with uranyl acetate and lead citrate and examined with a Hitachi H-600 transmission electron microscope (Hitachi, Tokyo, Japan) and Hitachi S-520 scanning electron microscopy (Hitachi, Tokyo, Japan) operated at 75 kV at a magnification of 20,000×.

### Statistical analysis

The data are expressed as the mean ± SD or as a percentage. Normality was checked using the Kolmogorov-Smirnov test. Non-normal variables were log transformed before statistical analysis. The continuous variables between two groups were compared using Student's t test. For continuous variables of more than two groups, statistical analysis was performed by one-way ANOVA followed by the SNK-q post hoc test. The categorical variables between two or more groups were compared using the chi-squared test. Differences in survival curves were checked using the log-rank test. Statistical significance was accepted at *P* < 0.05, and SPSS version 22.0 was used to analyze the data.

## Results

### NETosis is associated with delayed wound healing in DFU patients

We evaluated the level of NETosis biomarkers in the bloodstream of patients with DFUs (DFU group, n = 70) compared with matched diabetic patients without DFUs [DM (diabetes mellitus) group, n = 50] and matched control subjects without diabetes [NDU (non-diabetic ulcers) group, n = 50]. The cohorts' baseline characteristics are summarized in Table S1. Circulating levels of NETs markers (cfDNA, CitH3, and nucleosomes) were significantly increased in DFU group compared with diabetic patients without DFU and the healthy controls (Fig. S1A). As shown in Fig. S1B, neutrophil elastase (NE), the prototypical NET marker, was found at significantly higher levels in wounds of DFUs than NDUs. The elastase levels were significantly higher in unhealed wounds than healed wounds. Patients with amputation and wound infection had significantly higher elastase levels than those without amputation and infection. Circulating levels of cfDNA, CitH3, and nucleosomes correlated positively with the DFU patients' DUSS and WIfI scores (Fig. S1C). Serum CitH3 and tissue elastase concentrations progressively increased according to the DUSS. Concentrations of CitH3 and elastase in patients with DUSS of 3-4 were significantly higher than those of patients with DUSS of < 3 (Fig. S1D). According to the Kaplan-Meier curves, the probability of spontaneous healing over time was higher in patients with below-median serum CitH3 and tissue elastase concentrations (Fig. S1E).

### Diabetes primes NET formation, EndMT, and Hippo-YAP pathway inhibition in human and mouse wounds

**Figure 1:**
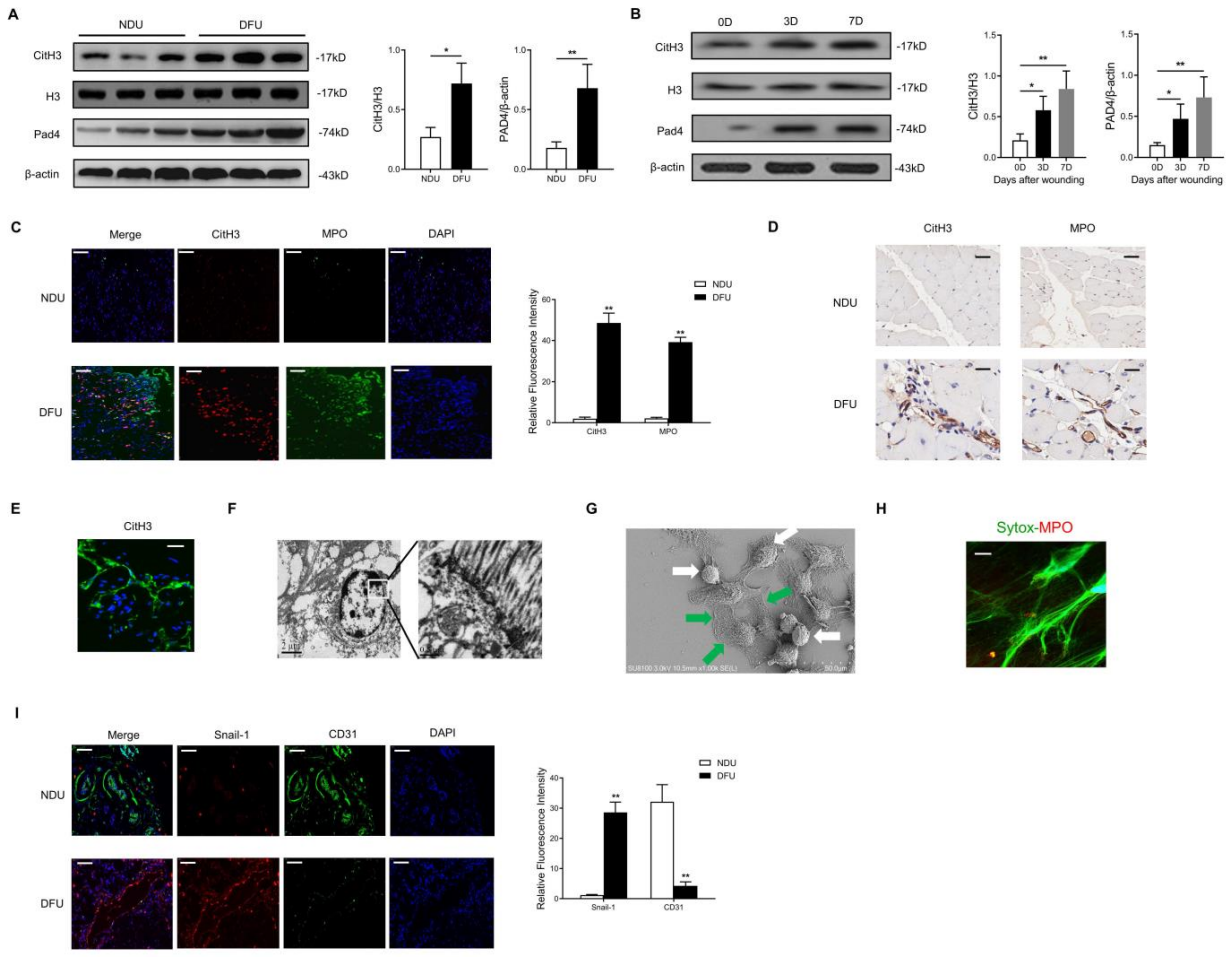
** Diabetes primes NET formation, EndMT, and Hippo-YAP pathway inhibition in human and mouse wounds.** (A) Western blotting analysis of typical NET marker, citrullinated histone H3 (Cit-H3), and protein arginine deiminase 4 (PAD4) protein levels in skin wounds from NDU vs DFU patients. Cit-H3 levels were normalized to histone H3 levels. PAD4 levels were normalized to β-actin levels. (B) Western blotting analysis of Cit-H3 and PAD4 in wound tissue at days 0, 3, and 7 post-ulcer formation in diabetic mice. Three independent samples from each group were analyzed. Cit-H3 levels were normalized to histone H3 levels. PAD4 levels were normalized to β-actin levels. (C-D) Immunofluorescence and immunohistochemistry images showing localization and expression of typical NET marker (CitH3, MPO) in wound tissue samples from NDU patients vs DFU patients, scale bar = 100μm in immunofluorescence images, scale bar = 1000μm in immunohistochemistry images. (E-F) High-resolution confocal immunofluorescence images (1000×, scale bar = 1μm) and transmission electron microscopy images (4000×, scale bar = 2μm; 20000×, scale bar = 0.5μm) of NETs formation in wound tissue of DFU patients. (G) Representative scan electron microscopy image of neutrophils and NETs in diabetic wound tissue. Green arrows point to extracellular meshes of NETs and white arrows point to neutrophils. (H) High-resolution confocal immunofluorescence images (1000×, scale bar = 1μm) of Sytox green and MPO dyeing in wound tissue. (I) Immunofluorescence images showing localization and expression of Snail-1 and CD31 in wound tissue samples from NDU patients vs DFU patients, scale bar = 100μm in immunofluorescence images. *P < 0.05, **P < 0.01, the variables between two groups were compared using Student's t test. For variables of more than two groups, statistical analysis was performed by one-way ANOVA followed by the SNK-q post hoc test. Data are shown as the mean ± SD, n = 6 in each group in this figure.

**Figure 2:**
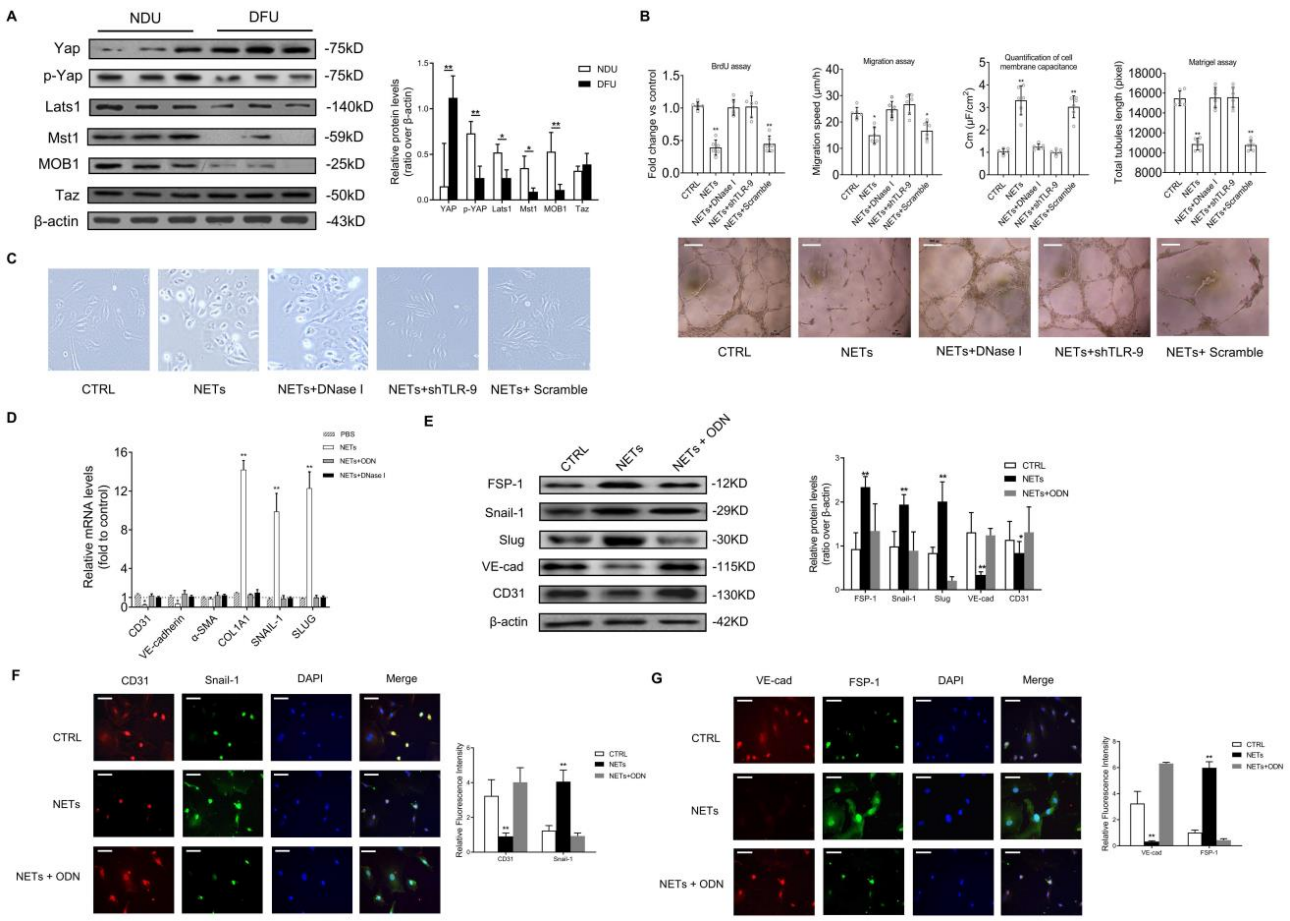
** NETs regulate EC functions and induce EndMT in a TLR-9-dependent manner.** (A) Western blotting analysis of the Hippo pathway (YAP, p-YAP, Last1, Mst1, MOB1, and Taz) in wound tissue from NDU vs DFU patients. β-actin is used as a loading control. (B) EC proliferation was analyzed by BrdU incorporation assays. EC migration was reported as migration speed (mm/h). Representative Matrigel assay images and quantification as total tubule length values are means ± SD. Cell membrane capacitance was also quantitatively compared. Scale bar = 500μm, (C) Morphological changes of ECs to mesenchymal phenotype were presented. (D) Relative mRNA levels of CD31, VE-cadherin, α-SMA, COL1A1, SNAIL-1, and SLUG. (E) Western blotting analysis of FSP-1, Snail-1, VE-cad, and CD31. β-actin is used as a loading control. (F-G) Immunofluorescence images showing localization and expression of Snail-1, CD31, VE-cad, and FSP-1. Scare bar = 50μm. DNase I, deoxyribonuclease I; ODN, oligonucleotide antagonist of the pattern recognition DNA receptor Toll-like receptor 9. *P < 0.05, **P < 0.01, the variables between two groups were compared using Student's t test. For variables of more than two groups, statistical analysis was performed by one-way ANOVA followed by the SNK-q post hoc test. Data are shown as the mean ± SD, n = 6 in each group in this figure.

The NET formation and Hippo-YAP pathway expression in diabetic wounds were evaluated. In contrast with elevated levels of CitH3 and Pad4 in the DFU wounds, low levels of CitH3 and Pad4 were detected in the NDU wounds (Fig. 1A). Similarly, high amounts of NETs were observed in the wound tissue of STZ-induced diabetic mice (Fig. 1B). In addition, the microenvironment (for example plasma and platelets) in the DFU patients was shown to prime neutrophils to release NETs (Fig. S2A-D). To investigate the NETosis and EndMT at the wound edge, the wounds were stained with CitH3, MPO, Snail-1, and CD31 antibodies. Confocal analysis showed increased expression of CitH3, MPO, and Snail-1 and decreased expression of CD31 in the wounds of the DFU patients compared with the NDU patients, consistent with the occurrence of NETosis and EndMT (Fig. 2C-D, 2I). High-resolution confocal immunofluorescence images of NET markers (Sytox green, CitH3, MPO) and transmission or scan electron microscopy images of NET formation in wound tissue of DFU patients were presented in Fig. 2E-H.

### NETs regulate EC functions and induce EndMT in a TLR-9 dependent manner

Compared with NDU wounds, the Hippo-YAP pathway in DFU wounds was found to be significantly inhibited (Fig. 2A). Then we explored the detailed molecular mechanism of this phenomenon. The DNA scaffold of NETs can be hydrolyzed by deoxyribonuclease I (DNase I) to short oligonucleotides 27. To analyze the effect of NETs on cell function of ECs, HUVECs were treated with NETs or DNase I-treated NETs. NET stimulation decreased the HUVECs' proliferation, migration, and tubulogenesis. The ECs' membrane capacitance was increased by NET treatment. The effect of NETs on cell function and morphology of HUVECs was no longer observed after cleaving NETs by DNase I (Fig. 2B-C). As the major NET components, histones and DNA are widely identified as endogenous damage-related molecular patterns that are recognized by TLR receptors 28. NETs prime macrophages for cytokine release in atherosclerosis via TLR 29. Thus, we hypothesized that the induction of EndMT was dependent on TLR. Significantly elevated mRNA and protein level of TLR-9 induced by NETs treatment in ECs was abolished by DNase I (Fig. S3A-B). TLR-9 loss of function with shRNA abolished the phenotypic effect of NETs on HUVECs, which indicated that NETs regulated EC functions via TLR-9 (Fig. 2B). During EndMT, loss of vascular endothelial cadherin (VE-cadherin) and platelet-EC adhesion molecule 1 (CD31) are always observed along with elevated expression of α-smooth muscle actin (α-SMA), vimentin, N-cadherin, and extracellular matrix proteins 30. NET stimulation increased the expression of collagen 1A1 (COL1A1), Snail-1, and SLUG, but decreased the expression of VE-cadherin and CD31, without significant changes in α-SMA (Fig. 2D). Moreover, expression analysis of the protein markers involved in EndMT showed strong upregulation of mesenchymal markers FSP-1, Snail-1, and Slug, along with significant suppression of CD31 and VE-cadherin in ECs receiving NET stimulation (Fig. 2E). Furthermore, immunofluorescence staining revealed that NET treatment resulted in a pronounced decrease in CD31 and VE-cadherin and an increase in Snail-1 and FSP-1 immunoreactivity. TLR-9 antagonist (ODN) or DNase I treatment can efficiently reverse NET-induced EndMT (Fig. 2F-G).

### NETs phosphorylate Merlin/NF2 and suppress the Hippo-YAP pathway in ECs

**Figure 3:**
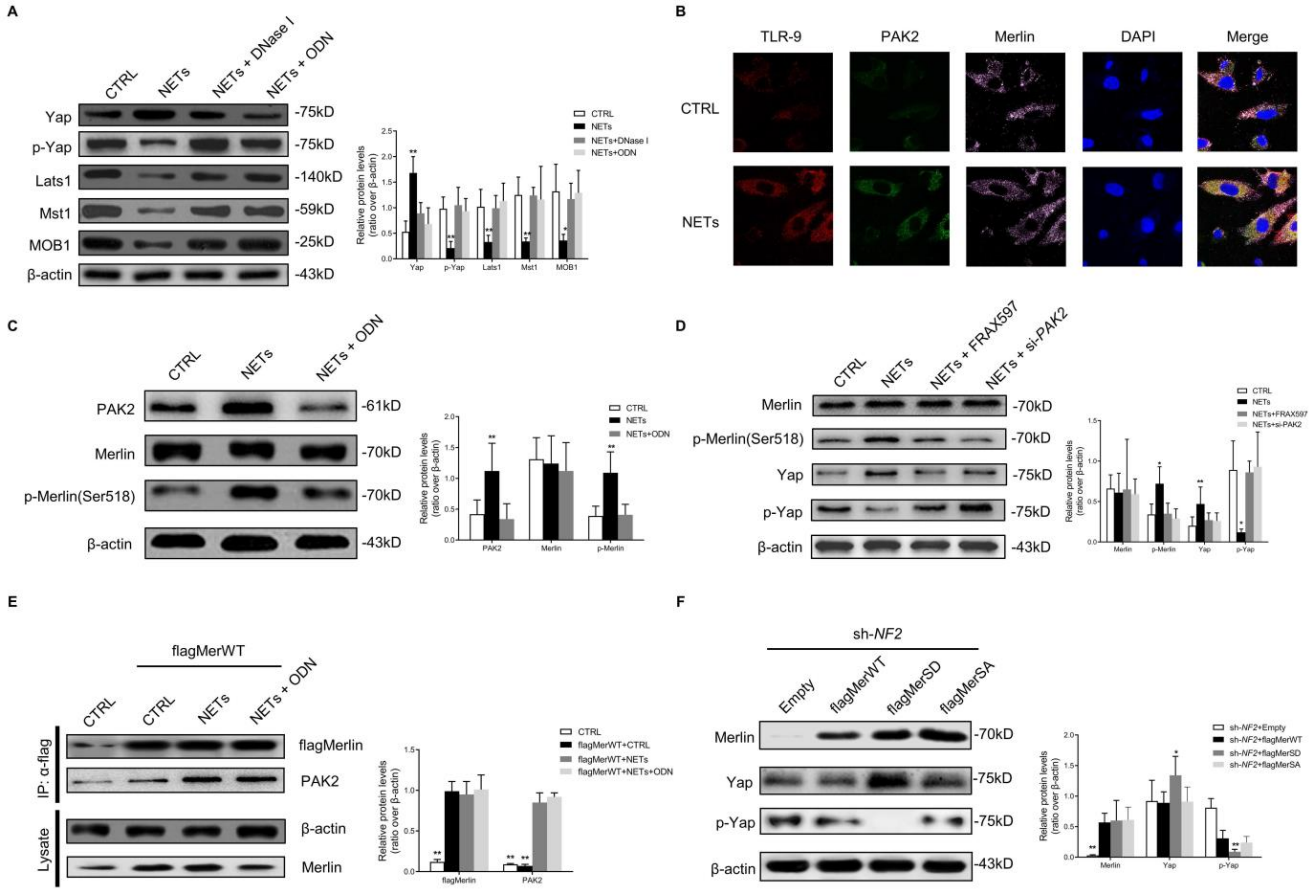
** NETs phosphorylate Merlin/NF2 and suppress the Hippo-YAP pathway in EC.** (A) Western blotting analysis of YAP, p-YAP, Last1, Mst1, and MOB1. β-actin is used as a loading control. (B) Immunofluorescence images showing localization and expression of TLR-9, PAK2, and Merlin/NF2 by triple dyeing. Scare bar = 20μm. (C) Western blotting analysis of PAK2, Merlin/NF2, and p-Merlin (Ser518). β-actin is used as a loading control. (D) Western blotting analysis of Merlin/NF2, p-Merlin (Ser518), YAP, and p-YAP. β-actin is used as a loading control. (E) Immunoblotting of HUVECs that were infected with lentivirus expressing the indicated flagMerWT constructs and immunoprecipitated with anti-flag antibody to detect the binding of endogenous PAK2 to flagMerWT constructs. In the control group, cells were infected with lentiviruses expressing empty vector. (F) Western blotting analysis of Merlin/NF2, YAP, and p-YAP. β-actin is used as a loading control. All the cells were infected with lentiviruses expressing shRNA of NF2. At 3 days post-lentivirus infection, cells were infected with lentiviruses expressing empty mCherry vector (Empty), flagMerWT, flagMerSA, or flagMerSD. Analysis was performed at 3 days post-lentivirus infection. FRAX597 is an ATP-competitive inhibitor of PAK2. The flagMerWT expresses the wild-type form of Merlin/NF2. The flagMerSA constitutively expresses a growth-inhibitory (activated) form of Merlin/NF2 in which there is an alanine substitution at the S518 phosphorylation site. The flagMerSD expresses a phosphomimetic (inhibited) form of Merlin/NF2. *P < 0.05, **P < 0.01, the variables between two groups were compared using Student's t test. For variables of more than two groups, statistical analysis was performed by one-way ANOVA followed by the SNK-q post hoc test. Data are shown as the mean ± SD, n = 6 in each group in this figure.

To investigate how NETs regulate the Hippo-YAP pathway in ECs, we firstly observed that NETs significantly suppressed the Hippo-YAP pathway in ECs, and ODN or DNase I treatment reversed the NET-induced suppression of the Hippo-YAP pathway (Fig. 3A). The microarray dataset (GSE189875) from the Gene Expression Omnibus (GEO) database was used for analysis. Gene set variation analysis (GSVA) based on curated gene sets revealed that endothelial cells treated with NETs display the activation of protein kinase associated pathway (Fig. S4A). The expressions of p21 activated kinase 2 (PAK2) demonstrated consistent and stable elevation in NETs treated endothelial cells (Fig. S4B-C). Based on protein-protein interaction analysis on STRING database, 16 upregulated genes exhibited potential interaction with PAK2. Then we ranked these 16 genes by using Diffusion, a network propagation algorithm on Cytoscape, and NF2 (encoding Merlin) was identified (Fig. S4D-E). The immunofluorescence images showed colocalization of TLR-9 and PAK2 at the HUVECs' membranes. PAK2 was colocalized with Merlin/NF2 in the cytoplasm with NET stimulation (Fig. 3B). The expression of PAK2 was strongly upregulated, and Merlin/NF2 was phosphorylated at S518 without significant change in its protein level after NET treatment. These changes were clearly reversed by TLR-9 antagonist (Fig. 3C). NETs-induced Merlin/NF2 phosphorylation and YAP dephosphorylation was inhibited by PAK2 loss of function with inhibitor or siRNA (Fig. 3D). The immunoprecipitation analysis demonstrated that NETs promoted wild-type Merlin/NF2 to bind PAK2 via TLR-9 (Fig. 3E). No direct interaction between TLR-9 and Merlin/NF2 was detected (Fig. S5A). After TLR-9 knockout, the direct binding of wild-type Merlin/NF2 to PAK2 was significantly weakened (Fig. S5B). Only phosphorylated Merlin/NF2 induced YAP dephosphorylation (Fig. 3F). These findings suggested that NETs phosphorylated Merlin/NF2 via TLR-9 and PAK2 to inhibit the Hippo-YAP pathway in ECs.

### YAP interacts with SMAD2 and promotes its translocation into the nucleus to induce EndMT

Global gene expression analysis and comparative transcriptome profiling was performed using wound tissue from patients with non-diabetic foot ulcers and infected or non-infected DFUs. By comparing differentially expressed genes from three groups with candidate transcription factors (TFs) from the TRANSFAC database, 93 differentially expressed TFs were identified (Fig. S6A). We used the Human Protein Reference and BioGRID databases to obtain the protein-protein interaction network structure from the query results (Fig. S6B). Four target TFs that play central roles in these protein-protein interactions were found. Among 4 TFs, expression level of *Smad*2 was significantly increased (Fig. S6C). Finally, SMAD2 was identified as a key TF in delayed healing of DFUs. In addition, we found upregulated protein level of SMAD2 in skin wounds from DFU patients compared with healthy controls and NDU patients (Fig. S7A-B). The expression of SMAD2 in wound tissue from STZ-diabetic animal models was higher than that in non-diabetic and STZ-diabetic *Padi4*^-/-^ animal models (Fig. S7C-D). NETs stimulation induced the upregulation of SMAD2 in HUVECs (Fig. S7E).

**Figure 4:**
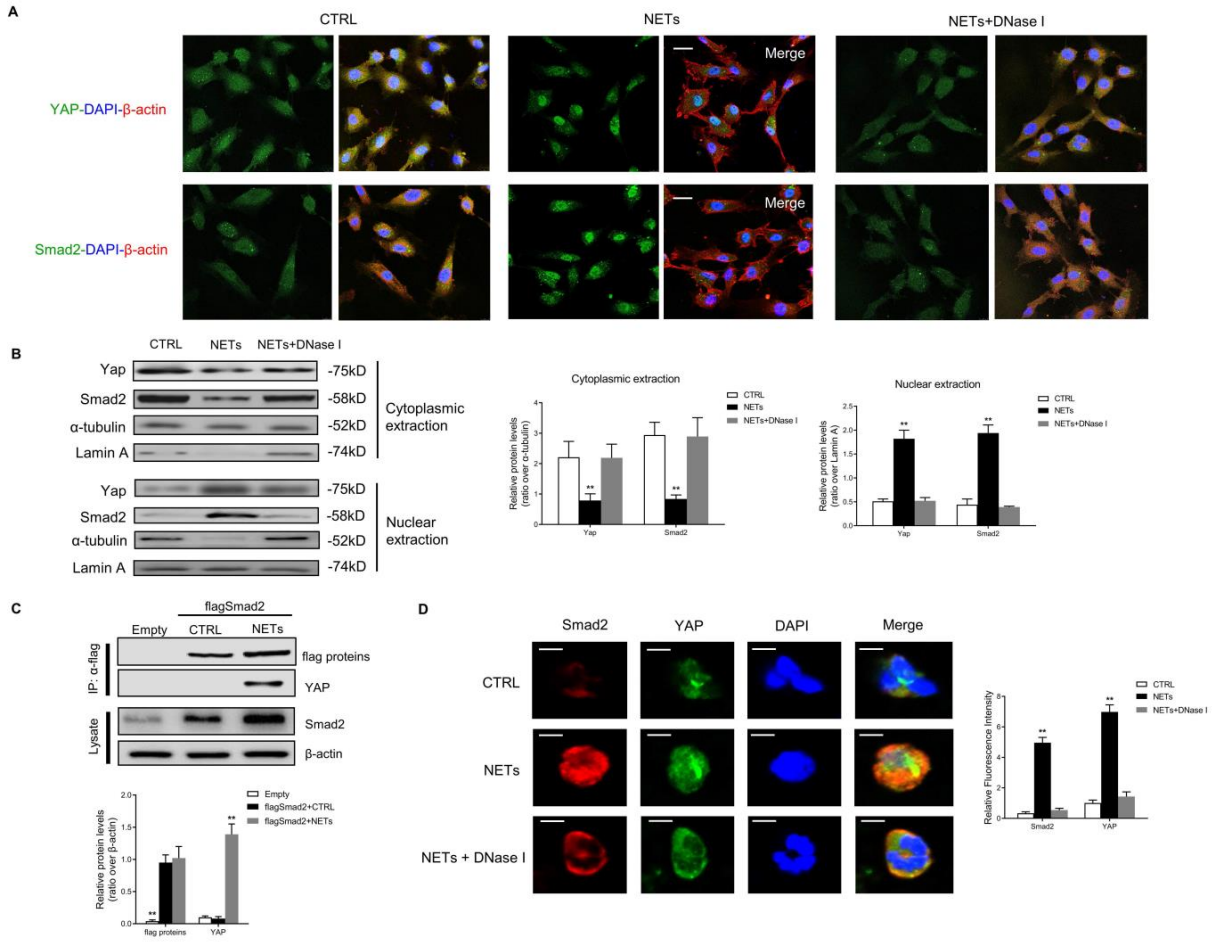
** YAP interacts with SMAD2 and promotes its translocation into the nucleus to induce EndMT.** (A) Immunofluorescence images showing the translocation of YAP and SMAD2 from the cytoplasm into the nucleus. The cytoskeletal staining was presented. (B) Western blotting analysis of YAP and SMAD2 in the cytoplasmic and nuclear fraction. Scare bar = 100μm. (C) Immunoblotting of HUVECs that were infected with lentivirus expressing the indicated flag SMAD2 constructs and immunoprecipitated with anti-flag antibody to detect the binding of endogenous YAP to flag SMAD2 constructs. In the control group, cells were infected with lentiviruses expressing the empty vector. (D) Immunofluorescence images showing colocalization and expression of SMAD2 and YAP in HUVECs. Scare bar = 20μm. *P < 0.05, **P < 0.01, the variables between two groups were compared using Student's t test. For variables of more than two groups, statistical analysis was performed by one-way ANOVA followed by the SNK-q post hoc test. Data are shown as the mean ± SD, n = 6 in each group in this figure.

Using the String Database, YAP was predicted to directly bind and was closely related to SMAD2 protein (Fig. S8). Then we investigated the binding and co-translocation of YAP and SMAD2 *in vitro* and *in vivo*. Immunofluorescence images showed the colocalization and co-translocation of YAP and SMAD2 from the cytoplasm into the nucleus after NET treatment (Fig. 4A). The cytoplasmic and nuclear fractions were extracted, and immunoblotting analysis revealed the co-translocation of YAP and SMAD2 (Fig. 4B). Their translocation was impeded with NET elimination by DNase I. Immunoprecipitation analysis confirmed the association between Merlin/NF2 and SMAD2 in HUVEC after NET treatment (Fig. 4C). Immunofluorescence staining displayed the NET-induced colocalization and upregulation of SMAD2 and YAP in the HUVECs (Fig. 4D).

### Suppression of Merlin/YAP/SMAD2 pathway attenuates NET-induced EndMT

**Figure 5:**
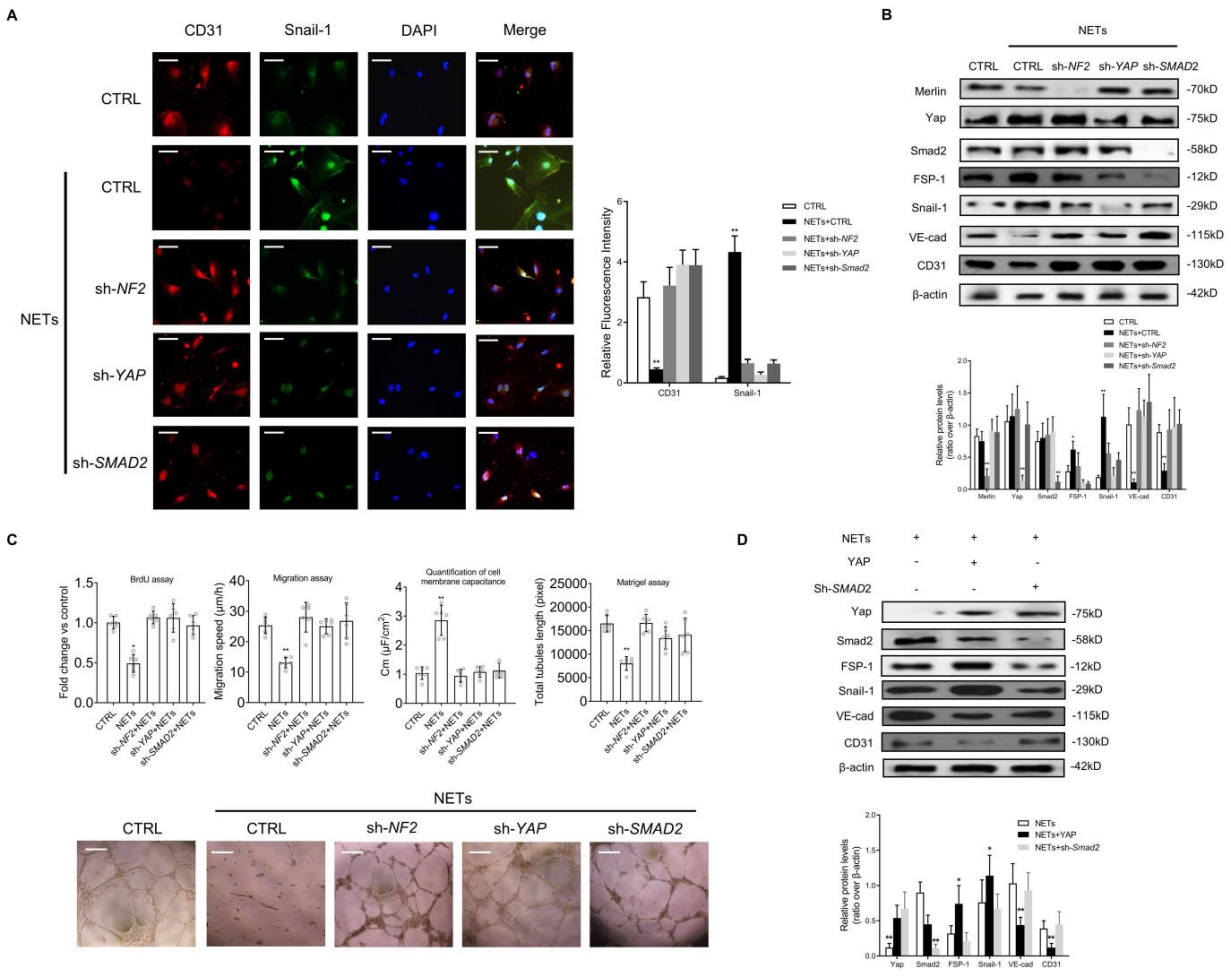
** Suppression of the Merlin/YAP/SMAD2 pathway attenuates NET-induced EndMT.** (A) Immunofluorescence images showing the localization and expression of Snail-1 and CD31. Scare bar = 100μm. (B) Western blotting analysis of Merlin/NF2, YAP, SMAD2, FSP-1, Snail-1, VE-cad, and CD31. β-actin is used as a loading control. (C) EC proliferation was analyzed by BrdU incorporation assay. EC migration was reported as migration speed (mm/h). Representative Matrigel assay images and quantification as total tubule length values are means ± SD. The cell membrane capacitance was also quantitatively compared. Scale bar = 500μm. (D) Western blotting analysis of YAP, p-YAP, SMAD2, FSP-1, Snail-1, VE-cad, and CD31. β-actin is used as a loading control. *P < 0.05, **P < 0.01, the variables between two groups were compared using Student's t test. For variables of more than two groups, statistical analysis was performed by one-way ANOVA followed by the SNK-q post hoc test. Data are shown as the mean ± SD, n = 6 in each group in this figure.

To confirm whether Merlin/YAP/SMAD2 pathway played a significant role during NET-mediated EndMT in ECs, we interfered with NF2, YAP, or SMAD2 using shRNA. We found that NET-mediated EndMT was markedly attenuated with knockout of NF2, YAP, or SMAD2 by immunofluorescence staining and immunoblotting analysis (Fig. 5A-B). NET-induced decrease of cell proliferation, migration, and tubulogenesis and increase of membrane capacitance were abolished with knockout of NF2, YAP, or SMAD2 (Fig. 5C). The immunoblotting results revealed that YAP overexpression significantly enhanced NET-induced EndMT. However, in the absence of SMAD2, YAP could not promote EndMT after NET treatment in HUVECs (Fig. 5D).

### NETosis Inhibition and SMAD2 knockdown accelerate wound healing by reducing EndMT and promoting angiogenesis in a diabetic mouse model

**Figure 6:**
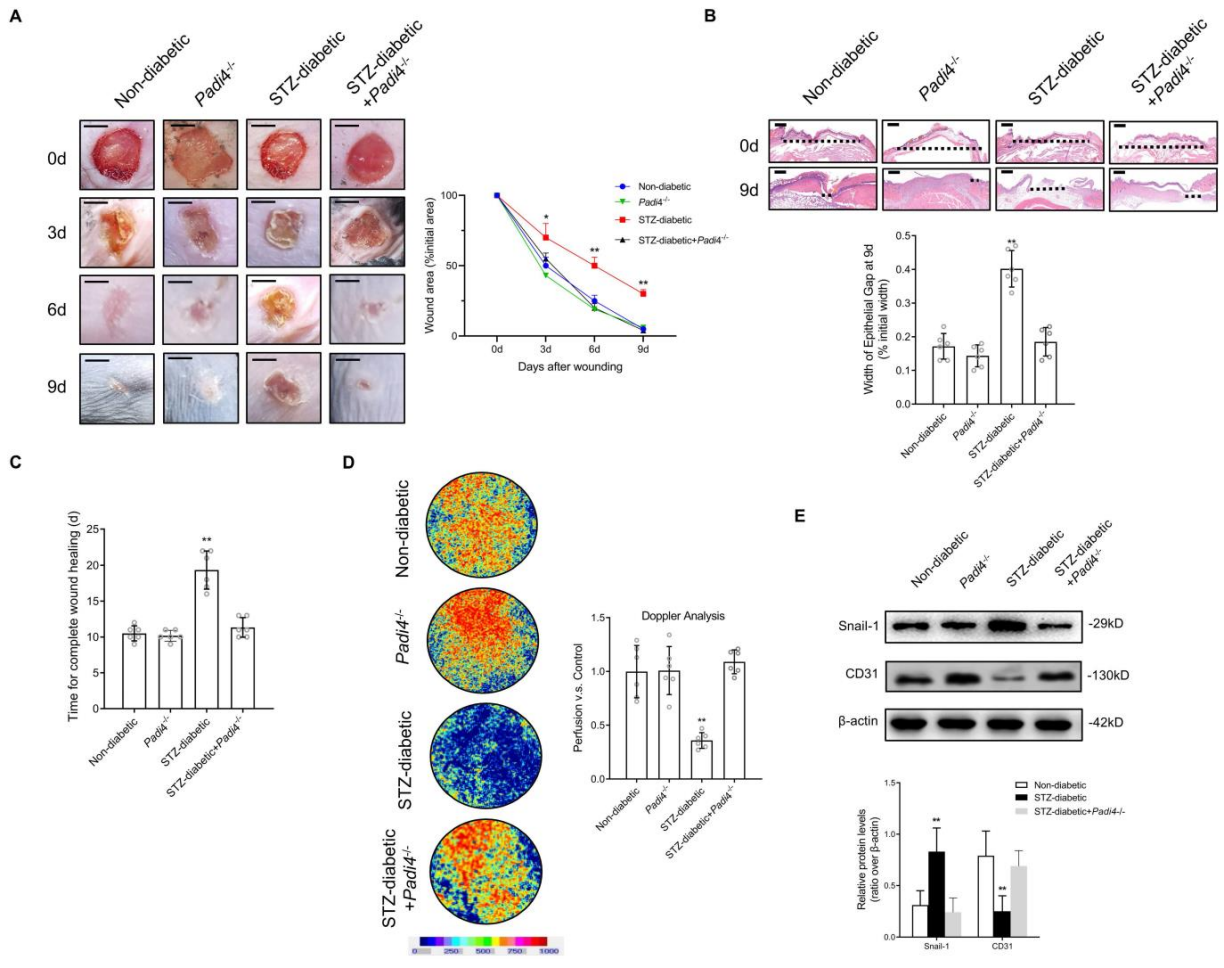
** NETosis Inhibition accelerate wound healing by reducing EndMT and promoting angiogenesis in a diabetic mouse model** (A) Left, representative images of treated wounds at 0, 3, 6, and 9 d post-wound injury in non-diabetic, STZ-diabetic, *Padi4*-/-, and STZ-diabetic + *Padi4*-/- groups. Right, level of wound closure is expressed as a percentage of wound area from the initial wound area. Scare bar = 500μm. (B) Epithelial gap of wound healing on histology was evaluated in non-diabetic, STZ-diabetic, *Padi4*-/-, and STZ-diabetic + *Padi4*-/- groups. The distance between the leading edges was calculated. Scare bar = 100μm. (C) Time for complete wound healing in different groups. (D) Representative color laser Doppler images taken at 5 days post-wounding in non-diabetic, STZ-diabetic, *Padi4*-/-, and STZ-diabetic + *Padi4*-/- groups. The chart shows the level of wound perfusion in mice (calculated as the ratio between treated and control blood flow). (E) Western blotting showing the upregulated expression of Snail-1 and downregulation of CD-31 in wound tissue from STZ-diabetic group compared with non-diabetic, *Padi4*^-/-^ or STZ-diabetic + *Padi4*^-/-^ groups. *P < 0.05, **P < 0.01, the variables between two groups were compared using Student's t test. For variables of more than two groups, statistical analysis was performed by one-way ANOVA followed by the SNK-q post hoc test. Data are shown as the mean ± SD, n = 6 in each group in this figure.

PAD4 is overexpressed in diabetes, and NETosis induction is demonstrated in murine models of DFU 31. Inhibition of NETosis by *Padi4* knockout or disruption of NETs with DNase I can accelerate diabetic wound healing 32. We investigated whether NETosis inhibition and SMAD2 knockdown can accelerate skin wound healing in a diabetic mouse model by suppressing EndMT. Macroscopic analysis and epithelial gap of wound healing on histology evaluation showed that wound closure was markedly improved in the STZ-diabetic model using *Padi4*^-/-^ mice (NETosis inhibition) in contrast to delayed wound healing in the STZ-diabetic model (Fig. 6A-B). The time for complete wound healing was significantly higher in the STZ-diabetic model than the non-diabetic and STZ-diabetic *Padi4*^-/-^ mouse models (Fig. 6C). To investigate NETosis and EndMT at the wound edge, the wounds were stained with Snail-1, CD31, CitH3, and MPO antibodies. Wound tissue perfusion was significantly impaired in the STZ-diabetic animal model compared with the non-diabetic model by Doppler analysis, and PAD4 knockdown also led to a significant increase in wound perfusion and vessel density (Fig. 6D and S9A). NETosis and EndMT were found to be significantly induced in the STZ-diabetic mouse model and knockdown of PAD4 *in vivo* suppressed NET-induced EndMT (Fig. 6E and S9B). Macroscopic analysis and epithelial gap of wound healing on histology evaluation also showed accelerated wound closure in the STZ-diabetic model with knockdown of SMAD2 in contrast to delayed wound healing in the STZ-diabetic model (Fig. S10A-B). Wound tissue perfusion was significantly reduced in the STZ-diabetic animal model compared with the non-diabetic model by Doppler analysis, and SMAD2 knockdown also led to a significant increase in wound perfusion and vessel density (Fig. S10C and S11A). Knockdown of SMAD2 *in vivo* also suppressed EndMT in the diabetic mouse model (Fig. S9D and S11B). These data indicated that NETosis inhibition and knockdown of SMAD2 can promote angiogenesis and accelerated wound healing by reducing EndMT *in vivo*.

**Figure 7:**
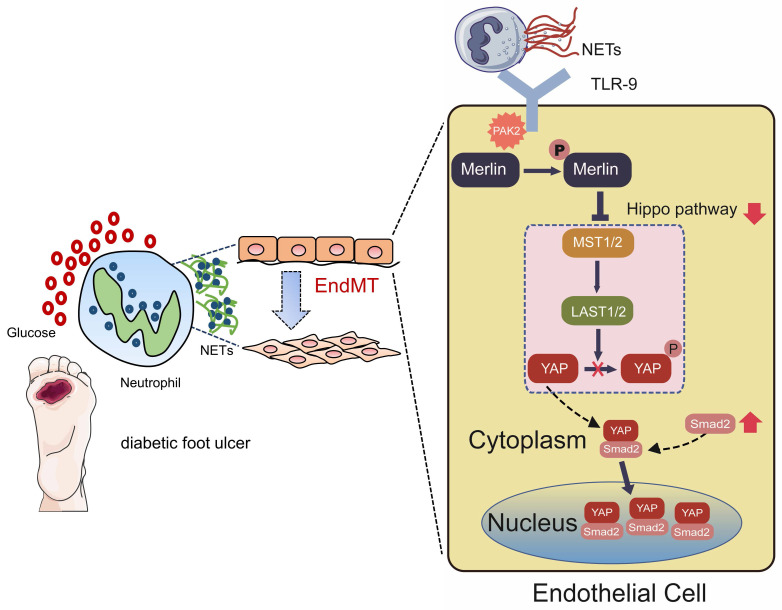
** Hypothetical model of NET participation in delayed healing of diabetic wounds by inducing endothelial-to-mesenchymal transition via the Hippo-YAP pathway.** Diabetic wound environment primes neutrophils to form NETs. NETs induce the activation of PAK2 via the membrane receptor TLR-9 in ECs. PAK2 phosphorylates the intracellular protein Merlin/NF2 to inhibit the Hippo-YAP signaling pathway. YAP binds to the transcription factor SMAD2. Then they translocate from the cytoplasm into the nucleus together to further induce the endothelial-to-mesenchymal transformation, which impedes angiogenesis and delays wound healing.

As shown in Fig. 7, in this study, we mainly found NET participation in delayed healing of diabetic wounds by inducing EndMT via the Hippo-YAP pathway. Moreover, our data suggested that inhibition of NETosis or suppression of Merlin/YAP/SMAD2 signaling axis can promote angiogenesis and accelerate wound healing by reducing EndMT. These findings will be of considerable value for providing cellular targets amenable to therapeutic intervention for DFUs.

## Discussion

It is reported that the finely tuned balance of NETosis seems to be lost in diabetes 33. In our previous studies, neutrophils were found to be primed to release NETs in patients with diabetes and sepsis 9, 34. In this study, neutrophils isolated from the peripheral blood of diabetic patients were found to have exaggerated NETosis response. NET components are enriched in non-healed wound of diabetic patients. The role of histones, cell-free DNA, and their combination in nucleosomes during NET-mediated wound healing delaying is unknown. TLR-9 is one of the primary membrane receptors for NETs to initiate intracellular signaling pathways. NETs-driven atherosclerosis and post-injury repair is mediated by TLR-9 29. Histones and phosphodiester DNA backbone, the major components of NETs, efficiently dimerize TLR-9, after which downstream inflammatory pathways are activated 35. TLR-9 knockout mice are protected from histone-mediated NLRP3 inflammasome activation during liver ischemia/reperfusion injury 36. These observations imply that histones and DNA act together to activate TLR-9.

In this study, we presented a mechanism that exaggerated NETosis delays the diabetic wound healing process by impairing angiogenesis. However, NETs are found to be multitasking in the regulation of angiogenesis. A functional link between NETs and inflammatory angiogenesis has been investigated in pulmonary arterial hypertension and thromboembolic pulmonary hypertension. NETs can stimulate EC proliferation, tube-like formation, and increase proangiogenic factors release by activating the TLR-4/NF-κB pathway 37. Additionally, NETs contribute to cancer invasion and tumor-related angiogenesis through the production of matrix metalloproteinase-9, vascular endothelial growth factor, and hepatocyte growth factor in primary and metastatic sites 38. The multiple effects of NETs on angiogenesis may depend on different signaling pathways. As we know, this is the first study to indicate that NETs can inhibit angiogenesis in skin wounds via the Hippo pathway.

We demonstrated that NETs delay the healing of DFUs via inducing EndMT in a Hippo-dependent manner. Upon skin tissue damage, dermal fibroblasts are activated and start proliferating and depositing/remodeling extracellular matrix 39. The Hippo-YAP signaling pathway can regulate skin wound healing. In diabetic wounds, YAP expression is reduced in fibroblasts, which can be recapitulated in vitro when dermal fibroblasts are cultured under high glucose conditions 40. However, the role of the Hippo pathway in endothelial cells during diabetic would healing has not been clearly disclosed. The Hippo pathway negatively controls bone angiogenesis by limiting hypoxia-inducible factor signaling in ECs 41. Mitochondrial damage and cytosolic DNA sensor cGAS-STING-IRF3 signaling can induce Hippo-YAP dysregulation that leads to angiogenesis impairment and delayed wound healing in diabetes 16. Herein, we demonstrated a specific mechanism that the Hippo pathway mediated the EC dysfunction and regulated angiogenesis by inducing EndMT via the transcription factor SMAD2, which is a core component of the TGF-β signaling pathway. YAP is required to modulate the expression of TGF-β signaling pathway components during skin wound healing 42. Our data are consistent with several other studies that the Hippo/TGF-β/SMAD signaling axis may contribute significantly to the EndMT process 43.

Unlike other developmental pathways activated by relatively specific ligands, studies in the past decade have linked Hippo pathway to a diverse array of upstream signals 44. The tumor suppressor Merlin/NF2 was the first upstream regulator genetically linked to the Hippo kinase cascade in drosophila 45. Merlin/NF2 regulates the core Hippo pathway kinases Lats1/2 and Mst1/2. However, the upstream regulator of Merlin in the Hippo pathway remains unknown. An important finding of this study is that NETs suppress the Hippo pathway via phosphorylation of Merlin at Ser518 in ECs. A similar regulatory mechanism between Merlin and the Hippo pathway was reported in another study. Angiomotin binding releases auto-inhibition and promotes Merlin binding to Lats1/2. Phosphorylation of Ser518 outside the Merlin auto-inhibitory site prevents angiomotin from binding and thus inhibits Hippo pathway kinase activation 15.

EndMT can lead to ECs acquiring a variety of different mesenchymal fates through different stages of differentiation 46. Previous studies reported the importance of EndMT in dermal fibrosis, showing that delay in wound closure is associated with excessive collagen deposition 47. Instead of secreting large amounts of collagen and other extracellular matrix proteins, the acquisition of a mesenchymal phenotype due to inflammation by ECs is likely to lead to endothelial dysfunction, thereby causing angiogenesis impairment 11. In this study, we found that NET-mediated EndMT had a significantly detrimental influence on angiogenesis and wound perfusion. The skin wound healing model seems to be an excellent way to assess the potential impact of EndMT on angiogenesis during the wound healing process. Excisional wounds rapidly close with active tissue remodeling of the wound area in a reproducible way. The use of an endothelial lineage-tracing mouse model during wound healing has highlighted the critical role of EndMT during skin wound healing 48. However, DFU is mainly a problem with type 2 diabetes in clinics. It could be a limitation that the STZ-induced diabetic mouse model in this study, which is widely used to study the NETs induced diabetic wound healing delay, is a type 1 diabetic mouse model 31, 32, 49. In addition, although the effect of microvascular endothelial cell in wound tissue healing under regulation of NETs has been shown in this study, the possibility that the effects in wound tissue are also due to changes in other cells cannot be ruled out since we use a global knockout mouse.

Increased understanding of the molecular mechanisms that regulate EndMT will be of considerable value for providing cellular targets amenable to therapeutic intervention for DFUs. The PKC βII inhibitor, ruboxistaurin, can reverse endothelial progenitor cell dysfunction and prevent exaggerated NET formation, thereby accelerating the wound healing 50. Hydrogen sulfide attenuates NETosis and primes diabetic wounds to heal through blockage of reactive oxygen species-mediated MAPK ERK1/2 and p38 activation 51. In addition, a novel scaffold system has been developed to deliver PAD4 inhibitor that can be used to modulate NETosis and improve diabetic wound healing 52. The therapeutic administration of DNase I can alleviate host injury in inflammatory or immunological conditions 53. DNase I was recently approved by the FDA for the treatment of cystic fibrosis (https://www.drugs.com/pro/pulmozyme.html) and can potentially translate into a stimulator of diabetic wound healing in the future.

In conclusion, this study illustrates a novel mechanism whereby NETs may delay diabetic wound healing by inducing EndMT via the Hippo-YAP pathway. The diabetic wound environment primes neutrophils to form NETs that induce PAK2 activation via TLR-9 in ECs. Then PAK2 phosphorylates the intracellular protein Merlin/NF2 to inhibit the Hippo-YAP pathway. YAP binds to SMAD2 and they translocate from the cytoplasm into nucleus together to promote EndMT, which ultimately impedes angiogenesis and delays diabetic wound healing. These findings suggest NETs inhibitors may have potential therapeutic effect for accelerating wound healing of DFU.

## Supplementary Material

Supplementary figures and tables.
