# Supplementary material for: Neutrophil Extracellular Traps Delay Diabetic Wound Healing by Inducing Endothelial-to-Mesenchymal Transition via the Hippo pathway
Source: Int J Biol Sci. 2023 Jan 1;19(1):347–61. doi: 10.7150/ijbs.78046 (PMC9760440; doi:10.7150/ijbs.78046)
Supplement: Supplementary file 1 — Supplementary figures and tables. [file ijbsv19p0347s1.pdf]

Table S1. Clinical and wound characteristics of all patients

Characteristic	CTRL (n = 50)	DM (n = 50)	NDU (n = 50)	DFU (n = 70)
**Age, y**	50.8 ± 9.5	65.8 ± 9.7	56.2 ± 10.6	71.2 ± 10.6
**Male sex, n (%)**	24 (48.0)	25 (50.0)	23 (46.0)	34 (48.6)
**Hemoglobin A_{1c}, n (%)**				
**Baseline**	/	7.3 ± 1.5	/	7.9 ± 1.2
**Nadir**	/	6.1 ± 1.9	/	6.7 ± 1.4
**Comorbidities, n (%)**				
**Hypertension**	28 (56.0)	23 (46.0)	13 (26.0)	46 (65.7)
**Dyslipidemia**	20 (40.0)	22 (44.0)	12 (24.0)	33 (47.1)
**Coronary artery disease**	23 (46.0)	19 (38.0)	15 (30.0)	35 (50.0)
**Congestive heart failure**	5 (10.0)	6 (12.0)	2 (4.0)	26 (37.1)
**Peripheral arterial disease**	4 (8.0)	8 (16.0)	3 (6.0)	67 (95.7)
**Chronic kidney disease**	10 (20.0)	12 (24.0)	7 (14.0)	22 (31.4)
**Dialysis**	3 (6.0)	6 (12.0)	3 (6.0)	12 (17.1)
**Stroke**	20 (40.0)	19 (38.0)	10 (20.0)	30 (42.9)
**Smoking, n (%)**	25 (50.0)	24 (48.0)	20 (40.0)	35 (50.0)
**Medication use, n (%)**				
**Aspirin**	26 (52.0)	20 (40.0)	20 (40.0)	41 (58.6)
**Clopidogrel**	14 (28.0)	10 (20.0)	10 (20.0)	28 (40.0)
**Warfarin**	1 (2.0)	2 (4.0)	2 (4.0)	5 (7.1)
**Novel anticoagulants**	1 (2.0)	2 (4.0)	2 (4.0)	3 (4.3)
**Statin**	20 (40.0)	15 (30.0)	15 (30.0)	37 (52.9)
**Diabetic medication, n (%)**				
**Oral only**	/	35 (70.0)	/	31 (44.3)
**Insulin**	/	15 (30.0)	/	39 (55.7)
**Wound area, cm^2**	/	/	10.9 ± 5.4	14.2 ± 6.5
**Wound depth, cm**	/	/	1.2 ± 0.5	1.1 ± 0.6
**Infection, n (%)**	/	/	10 (20.0)	16 (22.9)
**Time from onset to assessment, weeks**	/	/		6.7 ± 3.7
**Revascularization approach, n (%)**				
**Endovascular**	/	/	/	64 (91.4)
**Open**	/	/	/	3 (4.3)
**DUSS score**	/	/	/	1.5 ± 1.2
**WIfI score**	/	/	/	2.1 ± 1.1

CTRL, control; DM, diabetes mellitus; NDU, non-diabetic ulcer; DFU, diabetic foot ulcer; DUSS, Diabetic Ulcer Severity Score; WIfI score, wound, ischemia, and foot infection score

Table S2. List of primer sequences and shRNAs used in this study

**Primer sequences for mutagenesis**	
**flagMerSA**	5'-ACTGACATGAAGCGGCTTGCAATGGAGATAGAGAAAGAA-3'
**flagMerSD**	5'-ACTGACATGAAGCGGCTTGACATGGAGATAGAGAAAGAA-3'
**Sequences of shRNAs expressed by lentiviral vectors**	
**sh*TLR-9***	5'-CACCGCTGCCCAAATCCCTCATATCCGAAGATATG AGGGATTTGGGCAGC-3'
**si*PAK2***	PAK2 siRNAs (Thermofisher Scientific, #AM16708, ID:110776)
**sh*NF2***	5'-GATCCCCTGGTCAACGGAGCATTGATACGTGTGCT GTCCGTATCAGTGCTTCGTTGGCCATTTTTGGAAAT-3'
**sh*YAP***	5'-GATCCCC GACATCTTCTGGTCAGAGAACGTGTGCT GTCCGTTCTCTGACCAGAAGATGTCTTTTTGGAAAT-3'
**sh*SMAD2***	5'-CACCGCAGAACTATCTCCTACTACTCGAAAGTAGT AGGAGATAGTTCTGC-3'
**si*SMAD2***	SMAD2 siRNAs (Thermofisher Scientific, #AM16708, ID:107873)

Table S3. Blood glucose measurements of the mice in each group of Fig. 7

Groups (n=6)	0d, mmol/L	3d, mmol/L	6d, mmol/L	9d, mmol/L
**Non-diabetic**	5.48 ± 1.09	5.83 ± 0.97	5.62 ± 1.06	4.92 ± 1.06
***Padi*4**	4.89 ± 1.03	4.67 ± 0.90	4.37 ± 0.84	4.12 ± 1.05
**STZ-diabetic**	14.9 ± 1.20	14.6 ± 1.03	15.2 ± 0.81	14.6 ± 1.10
**STZ-diabetic + *Padi*4**	14.8 ± 1.14	15.3 ± 1.13	13.9 ± 0.94	15.2 ± 0.80
**STZ-diabetic + sh-*SMAD*2**	15.3 ± 1.04	14.7 ± 1.11	15.6 ± 1.17	14.6 ± 0.88
**STZ-diabetic + Scramble**	16.4 ± 0.94	15.1 ± 0.82	14.4 ± 0.89	15.6 ± 0.87

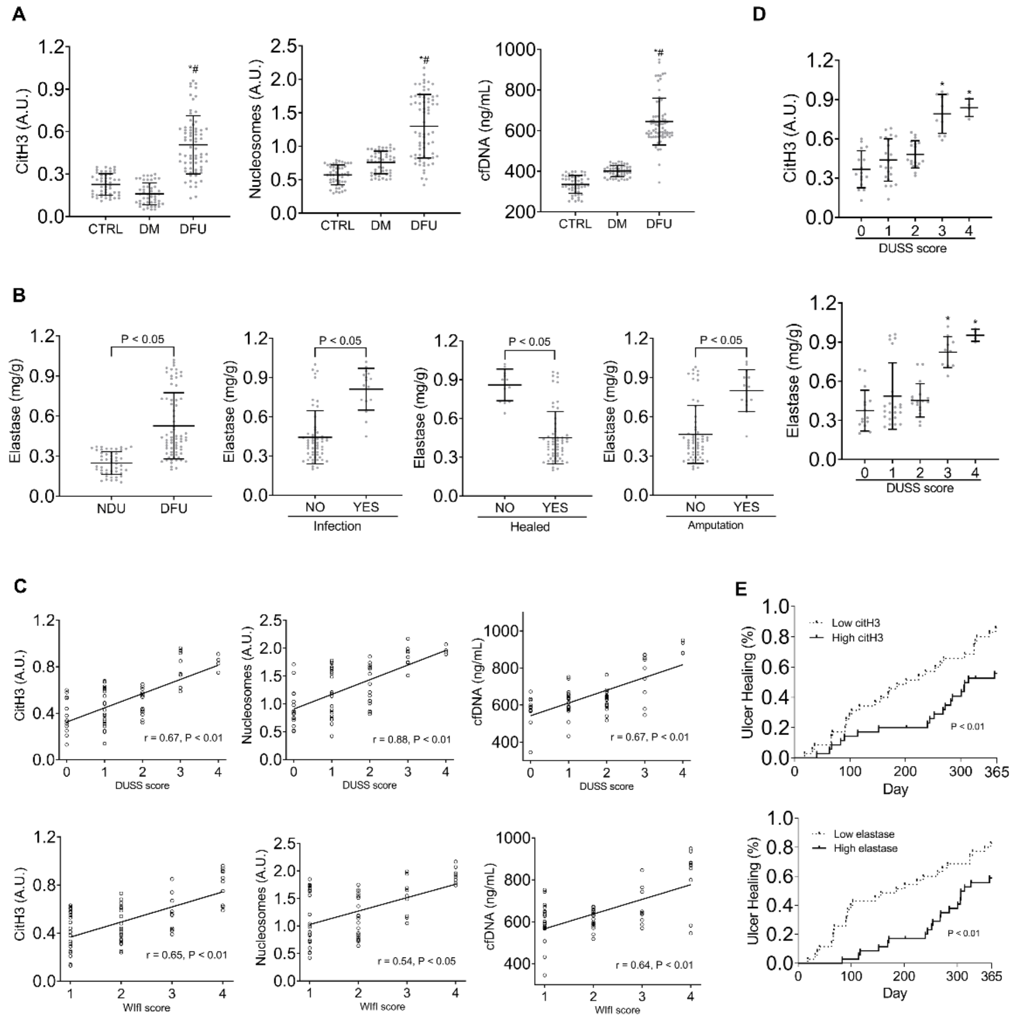

Supplemental Figure 1. NETosis is associated with delayed wound healing in DFU patients. (A) Levels of circulating NETosis biomarkers in patients with DFU, including CitH3, cfDNA, and nucleosomes in patients with DFU were significantly higher than those in diabetic patients without foot ulcers and healthy controls. The results are expressed as the means ± standard deviations for each group, n = 50 in CTRL group, n= 50 in DM group, n = 70 in DFU group. **P* < 0.05 vs CTRL group, #*P* < 0.05 vs DM group using one-way ANOVA followed by the SNK-q post hoc test. (B) Levels of elastase in ulcer tissue were significantly higher in DFU patients than NDU patients. Levels of elastase were significantly higher in wounds healed than in wounds not healed within 6 months. Patients with amputation and wound infection had significantly higher elastase levels than patients without

amputation and infection, n = 50 in NDU group, n = 70 in DFU group, the variables between two groups were compared using Student's t test. (C) Circulating levels of cfDNA, CitH3, and nucleosomes correlate positively with the DUSS and WIfI scores of DFU patients, total n = 70. (D) Levels of CitH3 in plasma and elastase in wound tissue progressively increased according to the DUSS. Concentrations of CitH3 and elastase in patients with DUSS of 3-4 were significantly higher than those in patients with DUSS of < 3, total n = 70, $^*P < 0.05$ using one-way ANOVA followed by the SNK-q post hoc test. (E) Kaplan-Meier curves showing the probability of ulcer healing in patients categorized as having low (below median) or high (above median) serum CitH3 and tissue elastase concentrations, n = 70.

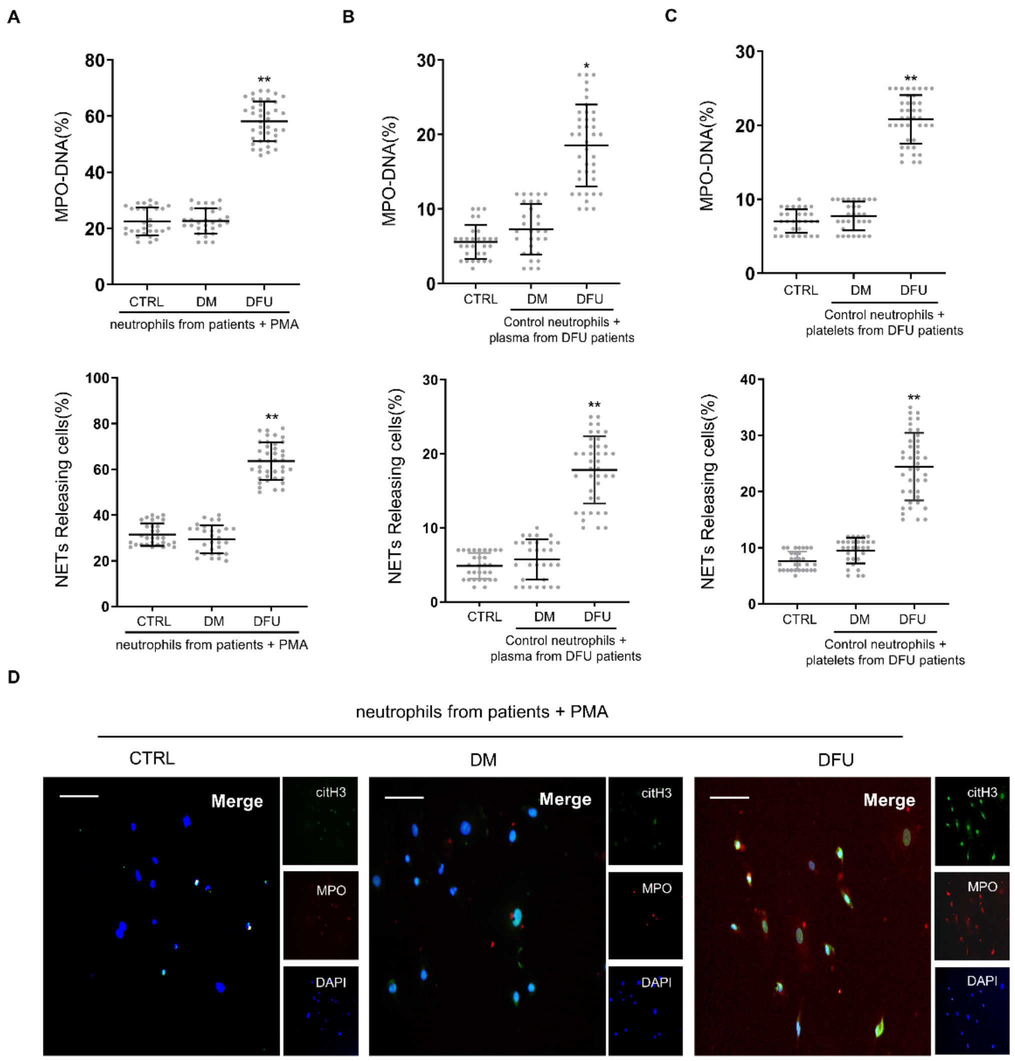

Supplemental Figure 2. The microenvironment in patients with DFU-primed neutrophils to release NETs. (A) Significantly increased levels of MPO-DNA complex and percentage of NET-releasing cells were observed in neutrophils from patients with DFU after PMA stimulation compared with neutrophils from healthy controls or diabetic patients without foot ulcers in vitro. n = 30 in CTRL group, n = 30 in DM group, n = 40 in DFU group. (B) Significantly increased levels of MPO-DNA complex and percentage of NET-releasing cells were observed in control neutrophils stimulated with plasma from patients with DFU compared with healthy controls or diabetic patients without foot ulcers in vitro. n = 30 in CTRL group, n = 30 in DM group, n = 40 in DFU group. (C) Significantly increased

levels of MPO-DNA complex and percentage of NET-releasing cells were observed in control neutrophils stimulated with platelets from patients with DFU compared with healthy controls or diabetic patients without foot ulcers in vitro. n = 30 in CTRL group, n = 30 in DM group, n = 40 in DFU group. For figure A-C, *$P < 0.05$ vs CTRL or DM group, **$P < 0.01$ vs CTRL or DM group using one-way ANOVA followed by the SNK-q post hoc test. (D) Representative microphotograph showing NETs releasing in neutrophils treated with PMA. The neutrophils are obtained from healthy controls, diabetic patients and DFU patients, respectively. Scale bar = 100μm.

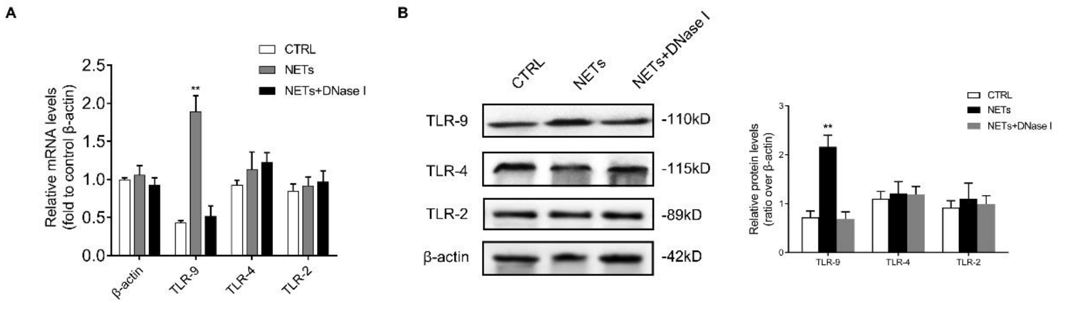

Supplemental Figure 3. NET stimulation led to elevated mRNA and protein levels of TLR-9 in ECs. Incubation of NETs with ECs led to elevated mRNA (A) and protein (B) levels of TLR-9, but not TLR-4 or TLR-2. n = 6 in each group, $^{**}P < 0.01$ vs CTRL or NETs + DNase I group using one-way ANOVA followed by the SNK-q post hoc test.

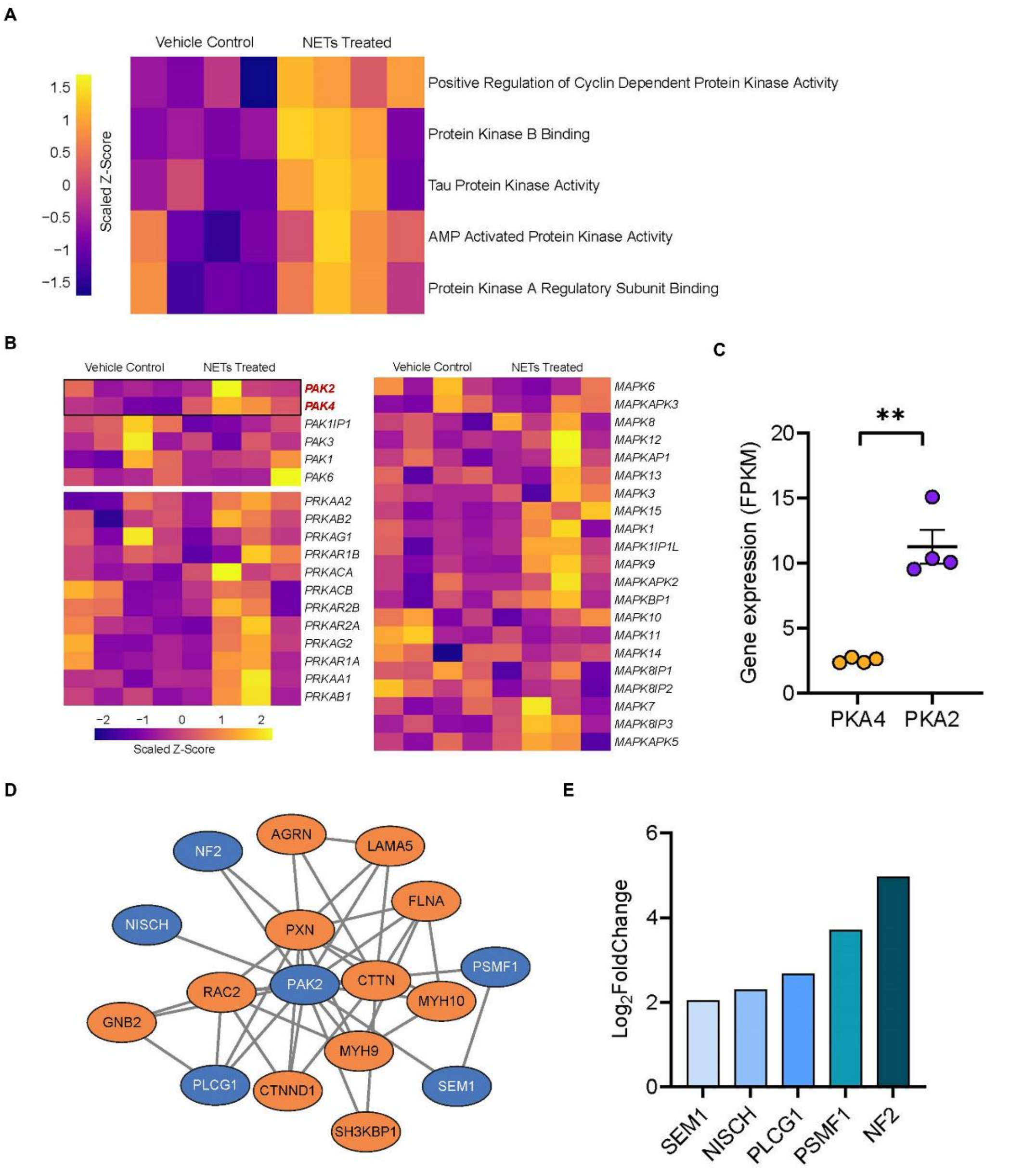

Supplemental Figure 4. (A) Gene set variation analysis (GSVA) based on curated gene sets (C5, which comprises the gene ontology terms associated with biological processes, molecular functions and cellular components) from molecular signatures database (MSigDB) revealed that endothelial cells treated with NETs display the activation of protein kinase associated pathway. (B) Within kinase encoding genes, the expressions of PAK2 and PAK4 demonstrated consistent and stable elevation in NETs treated endothelial cells. (C) The expression of PAK2 is much higher than PAK4. (D) 293 upregulated genes (log2FC>=2 and P<0.1) were first involved for protein-protein interaction analysis

on STRING database. 16 genes exhibited potential interaction with PAK2. Then we ranked these 16 genes by using Diffusion, a network propagation algorithm on Cytoscape, and identified top 5 genes (SEM1, NISCH, PLCG1, PSMF1, NF2). (E) Within above-mentioned top 5 genes, NF2 (encoding Merlin) exhibited the highest log2FoldChange. The microarray dataset (GSE189875) from the Gene Expression Omnibus (GEO) database was used for analysis.

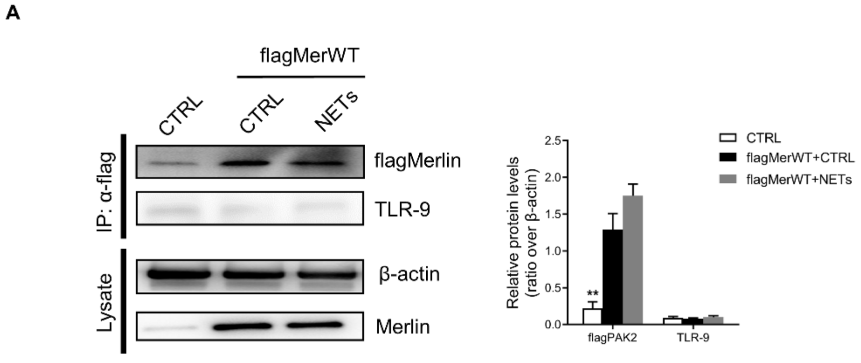

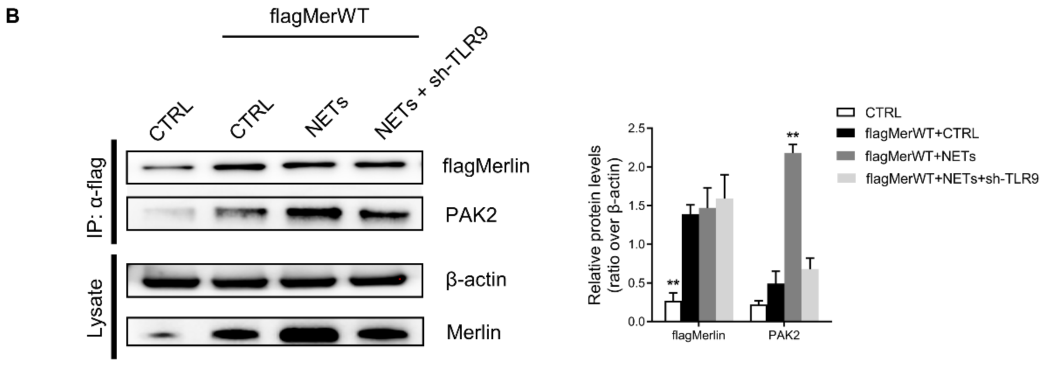

Supplemental Figure 5. (A-B) Immunoblotting of HUVECs that were infected with lentivirus expressing the indicated flagMerWT constructs and immunoprecipitated with anti-flag antibody to detect the binding of endogenous TLR-9 (A) or PAK2 (B) to flagMerWT constructs. In the control group, cells were infected with lentiviruses expressing empty vector. *$P < 0.05$, **$P < 0.01$, the variables between two groups were compared using Student's t test. For variables of more than two groups, statistical analysis was performed by one-way ANOVA followed by the SNK-q post hoc test. Data are shown as the mean ± SD, n = 6 in each group in this figure.

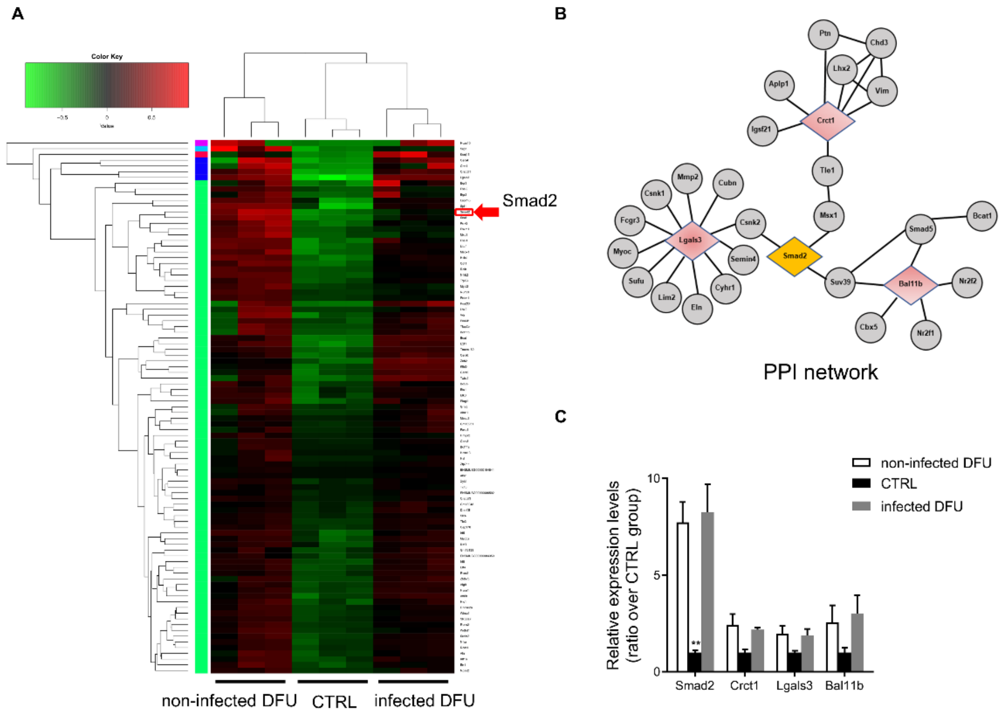

Supplemental Figure 6. (A) Global gene expression analysis was performed in the ulcer tissue from patients. Comparative transcriptome profiling was performed in the ulcer tissue from patients with non-diabetic foot ulcers (control) and infected or non-infected DFUs. By comparing differentially expressed genes from three groups (infected DFU vs control and non-infected DFU vs control) with candidate TFs from the TRANSFAC database, 93 differentially expressed TFs were identified. (B) We used both the Human Protein Reference (www.hprd.org) and BioGRID (www.thebiogrid.org) databases to obtain the protein-protein interaction (PPI) network structure from the query results. (C) Relative gene expression of 4 candidate TFs (Smad2, Crct1, Lgals3, Bal11b). $*P < 0.05$, $**P < 0.01$, the variables between two groups were compared using Student's t test. For variables of more than two groups, statistical analysis was performed by one-way ANOVA followed by the SNK-q post hoc test.

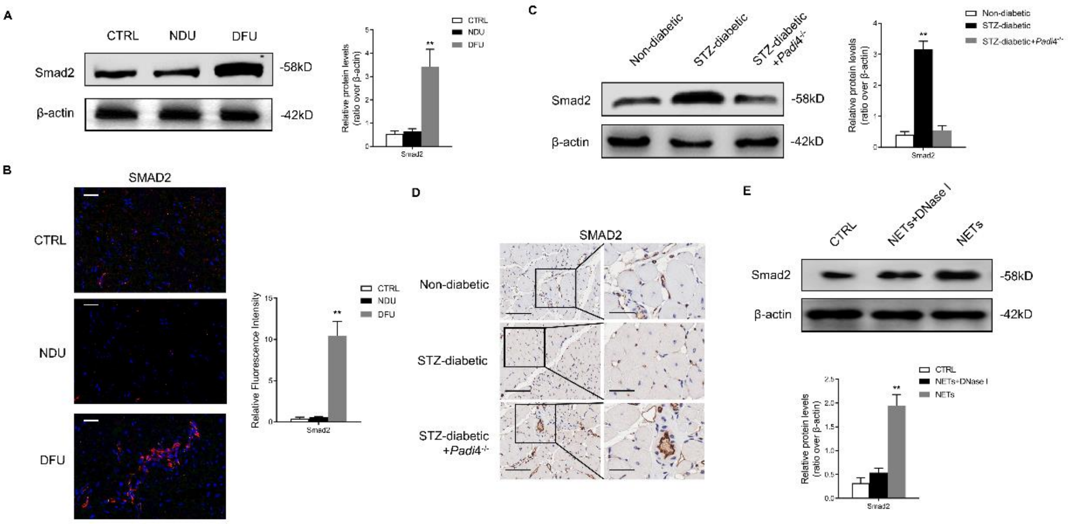

Supplemental Figure 7. (A-B) Western blotting and immunofluorescence staining showing the upregulated expression of SMAD2 in skin wounds from DFU patients compared with healthy controls and NDU patients. Scale bar = 100μm. (C-D) Immunoblotting and immunohistochemistry staining showed higher expression of SMAD2 in wound tissue from STZ-diabetic animal models than non-diabetic and STZ-diabetic *Padi4*$^{-/-}$ animal models. Left line: scale bar = 1000μm, right line: scale bar = 200μm. (E) Immunoblotting showed that NET stimulation induced the upregulation of SMAD2 in HUVECs. This effect was abolished with NET elimination by DNase I. $*P < 0.05$, $**P < 0.01$, the variables between two groups were compared using Student's t test. For variables of more than two groups, statistical analysis was performed by one-way ANOVA followed by the SNK-q post hoc test. Data are shown as the mean ± SD, n = 6 in each group in this figure.

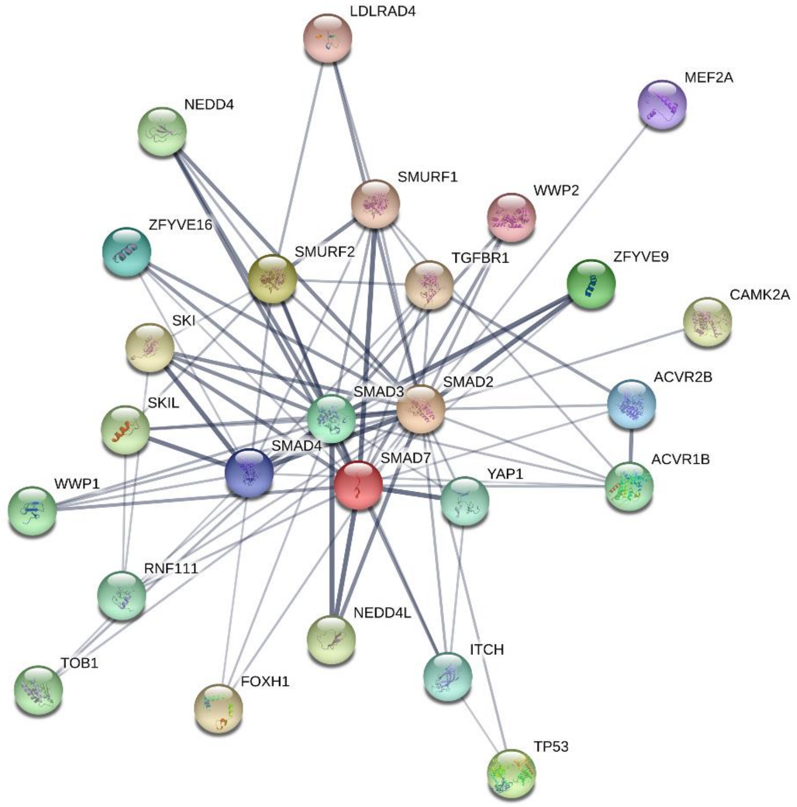

Supplemental Figure 8. YAP was predicted to directly bind and closely relate to SMAD2. Using the String Database, several proteins, including YAP, were predicted to directly bind and closely relate to SMAD2 protein.

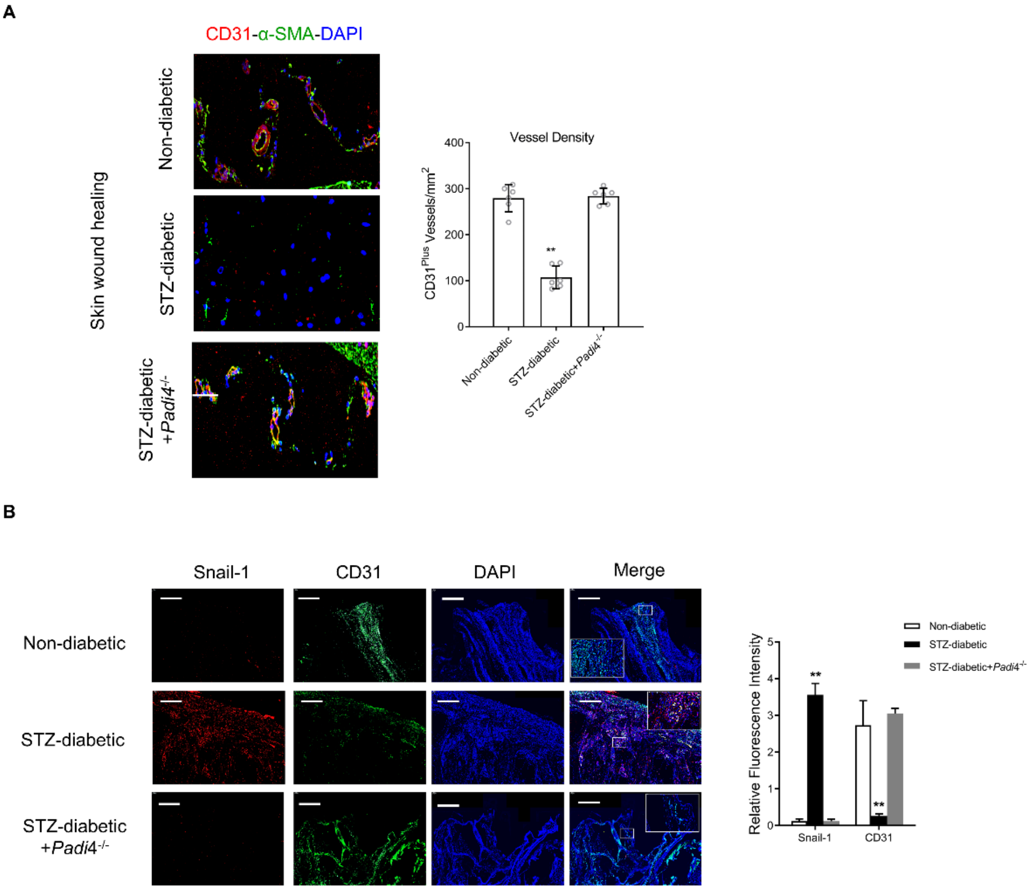

Supplemental Figure 9. PAD4 knockout can reduce NET-mediated EndMT and angiogenesis impediment. (A) Left, immunohistochemistry for CD31 and a-SMA in skin wounds. Right, quantification of vessel density expressed as CD31-positive vessels/mm^2. scale bar = 100μm. (B) immunofluorescence images showing localization and expression of EndMT markers (Snail-1, CD31). Scale bar = 500μm. Values are means ± SD. n = 6 in each group, *P < 0.05 and **P < 0.01 vs control using one-way ANOVA followed by the SNK-q post hoc test.

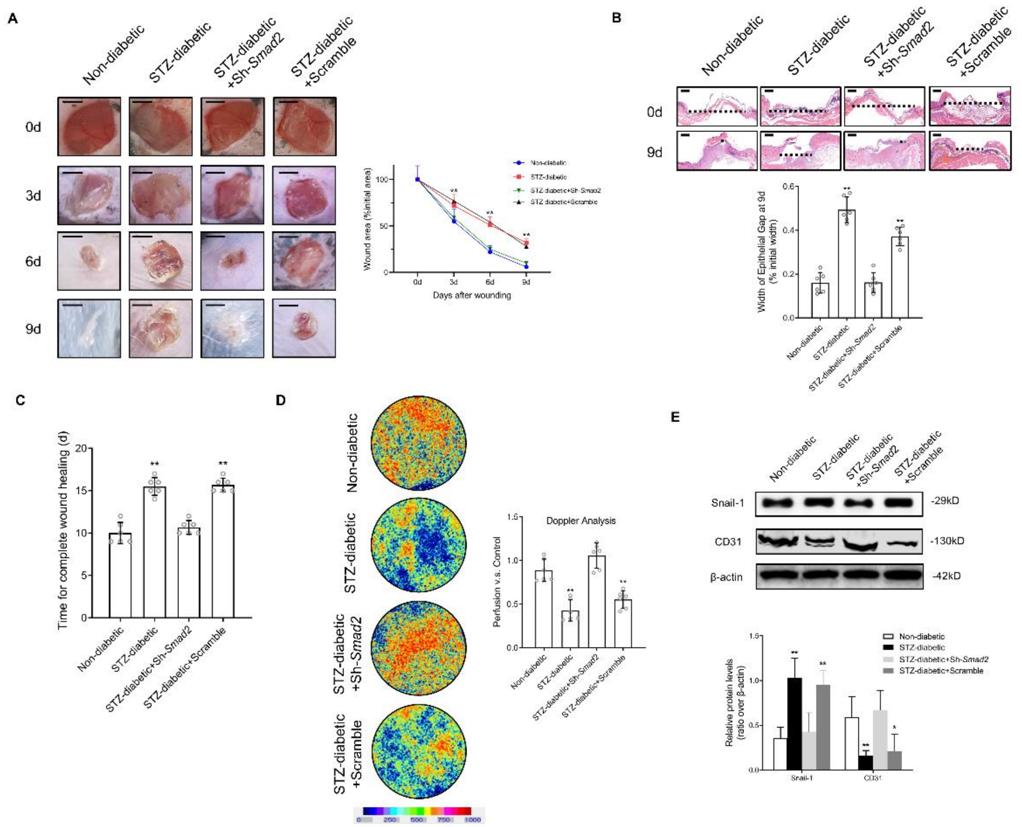

Supplemental Figure 10. *Smad*2 knockdown accelerate wound healing by reducing EndMT and promoting angiogenesis in a diabetic mouse model (A) Top, representative images of treated wounds at 0, 3, 6, and 9 d post-wound injury in non-diabetic, STZ-diabetic, Sh-*Smad2*, and STZ-diabetic + Scramble groups. Bottom, level of wound closure is expressed as a percentage of wound area from the initial wound area. Dermal wounds were treated with siRNA control oligonucleotides (Scramble) and *Smad2* siRNA for 9 days. Scare bar = 500μm. (B) Epithelial gap of wound healing on histology was evaluated in non-diabetic, STZ-diabetic, Sh-*Smad2*, and STZ-diabetic + Scramble groups. The distance between the leading edges was calculated. Scare bar = 100μm. (C) Time for complete wound healing in different groups. (D) Representative color laser Doppler images taken at 5 days post-wounding in non-diabetic, STZ-diabetic, Sh-*Smad2*, and STZ-diabetic + Scramble groups. The chart shows the level of wound perfusion in mice (calculated as the ratio between treated and control blood flow). (E) Western blotting showing the upregulated expression of Snail-1 and downregulation of CD-31 in wound tissue

from STZ-diabetic or STZ-diabetic + Scramble groups compared with non-diabetic or STZ-diabetic + Sh-*Smad2* groups. *$P < 0.05$, **$P < 0.01$, the variables between two groups were compared using Student's t test. For variables of more than two groups, statistical analysis was performed by one-way ANOVA followed by the SNK-q post hoc test. Data are shown as the mean ± SD, n = 6 in each group in this figure.

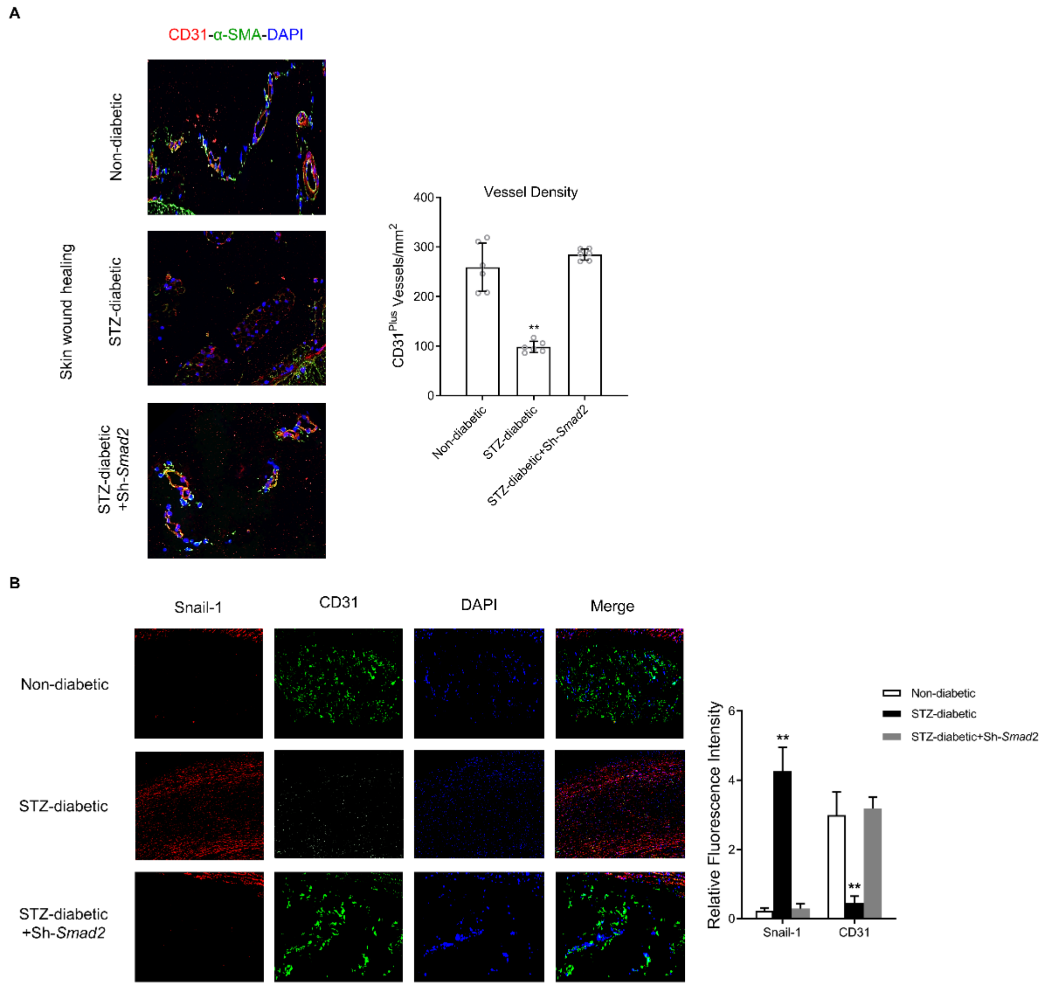

Supplemental Figure 11. *Smad*2 knockdown can reduce NET-mediated EndMT and angiogenesis impediment. (A) Left, immunohistochemistry for CD31 and a-SMA in skin wounds. Right, quantification of vessel density expressed as CD31-positive vessels/mm^2. scale bar = 100µm. (B) immunofluorescence images showing localization and expression of EndMT markers (Snail-1, CD31). Scale bar = 500µm. Values are means ± SD. n = 6 in each group in this figure, *$P < 0.05$ and **$P < 0.01$ vs control using one-way ANOVA followed by the SNK-q post hoc test.
